# Differential Dynamic Engagement within 24 SH3 Domain: Peptide Complexes Revealed by Co-Linear Chemical Shift Perturbation Analysis

**DOI:** 10.1371/journal.pone.0051282

**Published:** 2012-12-12

**Authors:** Elliott J. Stollar, Hong Lin, Alan R. Davidson, Julie D. Forman-Kay

**Affiliations:** 1 Program in Molecular Structure and Function, Hospital for Sick Children, Toronto, Ontario, Canada; 2 Department of Biochemistry, University of Toronto, Toronto, Ontario, Canada; 3 Department of Molecular Genetics, University of Toronto, Toronto, Ontario, Canada; Spanish National Cancer Center, Spain

## Abstract

There is increasing evidence for the functional importance of multiple dynamically populated states within single proteins. However, peptide binding by protein-protein interaction domains, such as the SH3 domain, has generally been considered to involve the full engagement of peptide to the binding surface with minimal dynamics and simple methods to determine dynamics at the binding surface for multiple related complexes have not been described. We have used NMR spectroscopy combined with isothermal titration calorimetry to comprehensively examine the extent of engagement to the yeast Abp1p SH3 domain for 24 different peptides. Over one quarter of the domain residues display co-linear chemical shift perturbation (CCSP) behavior, in which the position of a given chemical shift in a complex is co-linear with the same chemical shift in the other complexes, providing evidence that each complex exists as a unique dynamic rapidly inter-converting ensemble. The extent the specificity determining sub-surface of AbpSH3 is engaged as judged by CCSP analysis correlates with structural and thermodynamic measurements as well as with functional data, revealing the basis for significant structural and functional diversity amongst the related complexes. Thus, CCSP analysis can distinguish peptide complexes that may appear identical in terms of general structure and percent peptide occupancy but have significant local binding differences across the interface, affecting their ability to transmit conformational change across the domain and resulting in functional differences.

## Introduction

Specific protein∶ligand interactions are central to biological regulation and function and frequently involve changes in conformation and dynamics [1], [2]. Many proteins contain modular protein interaction domains, the roles of which are to specifically bind other proteins [3]. The most common of these domains in eukaryotes is the SH3 domain, which typically binds PxxPx+ or +xxPxxP consensus sites (where P is proline, + is a positively charged residue and x is any residue) [4]. One example is the SH3 domain from the yeast Actin Binding Protein 1 (Abp1p). Abp1p, in addition to its C-terminal SH3 domain, contains an N-terminal actin depolymerizing factor/cofilin homology (ADFH) domain and a central Pro-rich region. From its location in cortical actin patches, Abp1p helps coordinate the events culminating in endocytosis [5]. The Abp1p SH3 domain (AbpSH3) is central to the myriad of protein interactions involved in this coordination, making biologically relevant interactions with proline-rich disordered regions of the actin patch kinases, Ark1p and Prk1p [6], and the Synaptojanin-like protein, Sjl2p [7]. The importance of the AbpSH3 interactions in yeast and the high conservation of Abp1p homologues across species from fungus to humans inspired our previous structural, biophysical and functional investigations of this domain [8].

**Figure 1:**
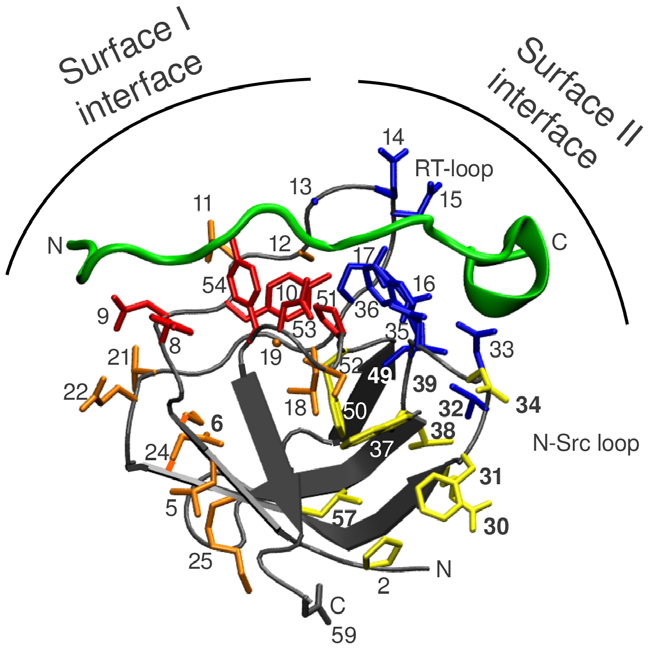
Structure of AbpSH3 (grey) bound to ArkA (green), emphasizing residues that undergo significant chemical shift changes on peptide binding. SI residues are colored red and residues associated with SI are colored orange (only the amide nitrogen of residue 19 is shown to avoid too much crowding). SII residues are colored blue and residues associated with SII are colored yellow. The N- and C-termini for protein and peptide are also indicated that includes the side-chain of asparagine 59 in AbpSH3. The labels for residues found to display co-linear chemical shift behavior in Fig. 2A are in bold. Chemical shift differences between peptide-free and ArkA-bound AbpSH3 can be found in Table S1.

AbpSH3 and nearly all other SH3 domains share a binding surface called surface I (SI), which is common amongst orthologs and paralogs (Fig. 1) and interacts with the PxxP motif. An adjacent surface called surface II (SII), which is variable amongst many yeast paralogs, binds sequences immediately N- or C-terminal to the PxxP motif [8], [9]. SII (also referred to as the specificity pocket) allows different SH3 domain paralogs to distinguish between peptide targets leading to higher specificity beyond the canonical PxxP motif recognized by SI [8], [10], [11]. Since many SH3 domain interactions involving both surfaces are mediated by extended peptide targets and flexible protein loops that can in theory be highly dynamic, we hypothesized that bound target peptides could exist in a dynamic equilibrium between fully engaged (where the complete binding interface is immobilized) and partially engaged conformations and that this equilibrium could be related to the structure, thermodynamics and function of the interaction. Since a large number of peptides that bind AbpSH3 have been characterized [8], we sought a rapid method to characterize any dynamics and conformational changes present in all of these complexes.

NMR spectroscopy is a powerful tool to address the above hypothesis and establish a method for examining dynamics in many complexes. Previous NMR studies measuring co-linear chemical shift (CCSP) behavior have revealed equilibrium distributions of protein conformations whose populations could be shifted by phosphorylation or mutation [12]–[22], although few studies have extended this technique to probe protein-protein or protein-peptide interactions. CCSP behavior is defined as the observation of a linear pattern of chemical shifts for resonances of the same residue in different related proteins (*i.e.* different mutants of the same protein or the same protein with different but related binding partners) when their 2D correlation spectra are overlayed. In general, when a series of perturbations are made to a protein the chemical shift changes for the same residue are not correlated. However, when a linear pattern of chemical shifts is observed, it definitively indicates that this residue exists in just two conformational states that have distinct chemical shift environments and are in fast exchange on the NMR timescale.

We demonstrate CCSP behavior in over a quarter of the AbpSH3 residues for 24 peptide complexes and characterize these complexes *in vitro* using NMR spectroscopy and isothermal titration calorimetry. We find clear evidence for significant dynamics in the bound state leading to partial engagement of the peptide, in contrast to the general view that modular binding protein∶peptide complexes fully engage their peptide targets. We observe that these dynamics correlate with thermodynamic data as well as functional data. Therefore, CCSP analysis provides a straightforward way to analyze large sets of protein∶peptide complexes which are in fast exchange to reveal their structural and functional properties. Our CCSP analysis of these SH3 domain complexes points to different local binding energies across a peptide-protein interface and highlights the importance of bound state dynamics to structure and function.

## Materials and Methods

### Sample preparation

The AbpSH3 (uniprot # P15891), ArkA (uniprot # C7GWN7) and L(−7)A peptides were expressed as thioredoxin fusions with a TEV cleavage site and were purified as in [8]. All other peptides were synthesized by Biomer tech (San Francisco) with N-terminal acetylation and C-terminal amidation and purified to greater than 95% by HPLC. Peptide concentrations were determined by amino acid analysis (Advanced Protein Technology Center, Hospital for Sick Children, Toronto).

### Thermodynamic measurements

Binding isotherms were recorded as previously at 30°C [23]. Enthalpy changes were measured at 4 different temperatures (20, 25, 30, 35°C) and the slopes of temperature vs. enthalpy change were fit to yield the change in heat capacity. All samples were in 50 mM sodium phosphate, 100 mM NaCl, 1 mM EDTA pH 7.0. Errors are ±95% confidence intervals except the ΔC_P_ values, which report the standard error of the least squared fit to the data (ΔH *vs.* temperature).

### NMR spectroscopy

NMR experiments were carried out at 30°C on Varian 500 MHz spectrometers equipped with pulsed field gradients and a triple resonance probe with actively shielded z-gradients. Data were processed and analyzed using nmrPipe/nmrDraw [24] and nmrView [25]. All spectra were recorded in 50 mM sodium phosphate, 100 mM NaCl, 1 mM EDTA pH 7.0. ^1^H-^15^N HSQC spectra were collected using standard methods [26]. ^1^H-^13^C HSQC spectra were recorded using constant time HSQC experiments [27].

AbpSH3 complexes were assigned using the established assignments of ArkA-bound and free AbpSH3 [8]; serial peptide additions were used to monitor the direction that the peaks moved to assign the final position of the SH3 resonances. Almost all of the peptide complexes are in fast exchange on the NMR timescale (for free to bound state inter-conversion) facilitating this method of assignment. The assignments were also compared to the previously determined ArkA-AbpSH3 complex resonances [8]. The assignments of the last 6 residues of the L(−7)A peptide were made by proximity to their ArkA counterparts [8] rather than independent triple resonance experiments. Only the final peptide-saturated complexes were used in the CCSP analysis.

In all cases 99% or greater peptide saturation was achieved; this was established either using peptide titrations until the resonances did not shift any further and/or adding sufficient peptide based on the known *K*_d_ value of the complex (the *K*_d_ values determined by NMR and ITC were almost identical, see Fig. S1 for more details). ^1^HN-^15^N NOE data were collected for the free AbpSH3, ArkA L(−7)A-bound and ArkA-bound complex using standard methods [28].

All hydrogen exchange experiments were conducted in 50 mM sodium phosphate, 100 mM NaCl, 1 mM EDTA pH 7.0, 18°C. Based on the known binding constants, sufficient peptide was added to 100 µM AbpSH3 such that 99% saturation was achieved (this was confirmed by comparing the chemical shifts of previously recorded peptide-saturated samples) for 7 different AbpSH3 complexes (Table S4). The samples were lyophilized and resuspended in D_2_O, and ^1^H-^15^N HSQC spectra were recorded every 20 minutes over a period of 24 hours. Many peaks disappeared over time. Intensities of peaks were fit to exponential curves, to yield observed rate constants (k_exchange_) for each amide group. The rate constant was converted to log protection factor (P), log_10_(k_intrinsic_/k_exchange_) using values for amide protection of free amino acids (http://hx2.med.upenn.edu/download.html). The log of amide protection factors were summed for residues 17, 32, 33 and 36 to represent the protection in SII (Σ LogP in SII). These residues became protected to different degrees in the different complexes although they all still exchanged sufficiently fast for their rates to be accurately calculated within the timeframe of the experiment. The biggest difference for the sum between complexes is about 20, which corresponds to a residue ΔΔG of −2.8 kcal/mol, which is approximately the difference between the strongest and weakest peptide binding energy.

### Calculation of the CCSP value

**Figure 2:**
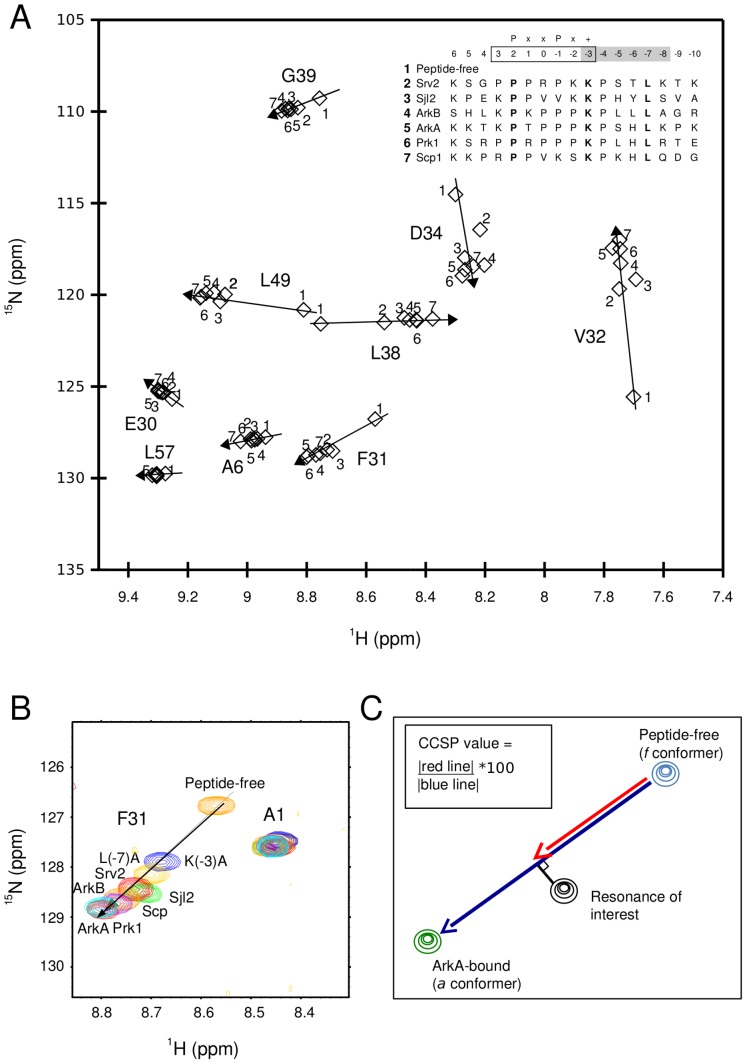
Co-linear chemical shift perturbation is found for 9 AbpSH3 residues in the initial set of 6 complexes. (A) Overlay of 7 NH correlation spectra (peak positions are represented by diamonds). Inset: an alignment of the AbpSH3 peptide targets, indicating the numbering system used for the peptides and the position of the PxxPx+ motif for class II binding peptides, where P is a proline, x is any residue and + is a positively charged residue. Residues 3 to −3 are part of the SI binding region (box) and residues −3 to −8 are part of the SII binding region (shaded grey). The residues in bold are the three most energetically important residues for ArkA binding to AbpSH3. (B) An overlay of the F31 and A1 backbone NH resonance set from spectra of all complexes from (A) plus 2 ArkA mutant complexes. Peptide-free is orange, K(−3)A is blue, L(−7)A is gold, Sjl2 is green, Srv2 is red, Scp1 is orange, ArkB is purple, ArkA is cyan and Prk1 is red. (C) CCSP calculations for an example NH group found in free AbpSH3 (blue peak), AbpSH3 bound to a variant peptide (black peak) and AbpSH3 bound to ArkA (green peak). The CCSP value for the resonance found in the complex between AbpSH3 and the variant peptide is calculated by dividing the length of the red line into the blue line (CCSP line). The black line is the perpendicular distance of the resonance to the CCSP line and is used to assess how close it is to the CCSP line.

In order to calculate a CCSP value, a CCSP line was defined between the resonance in the free AbpSH3 state with the resonance in the ArkA-bound AbpSH3 state (blue line in Fig. 2C). A line between the resonance of interest and the corresponding peptide-free resonance had its scalar projection onto the CCSP line (red line) calculated in the following way: scalar projection (s) = (z^2^+x^2^−y^2^)/(2z), where, z = length of the line connecting ArkA-bound resonance and peptide free resonance, y = length of line connecting resonance to ArkA-bound resonance, and x = length of line connecting resonance to peptide free resonance.

In choosing a bound state to define the CCSP line, we looked for the most stable complex that is most likely to represent the *a* conformer for residues affected by binding. Given that 15 out of the 24 complexes were ArkA mutants or truncations, the ArkA complex was deemed the most representative fully bound state to aid in our analysis. A scalar projection in combination with a measurement of the perpendicular distance of the resonance from the line was used so as to calculate CCSP values in reference to the CCSP line and to minimize errors associated with any small deviations from the line due to local effects other than the simple 2-state exchange of the residue-level conformers *f* and *a.* This approach is similar albeit more rigorous to the majority of studies analyzing CCSP values that interpret exchange at the residue level to occur between 2 pure protein conformations, allowing these 2 populations to be calculated [12]–[16], [18]–[20], [22]. This approach is different from a more recent study that calculated the absolute chemical shift changes in different complexes to look for correlations in residue populations without calculating the populations of protein-level conformations [21].

The combined ^1^H and ^15^N chemical shift differences (in ppm) between each pair of resonances that define the lines x, y and z were calculated using the following expression: δΔ = √((N_ppm_ shift * 0.1)^2^+(H_ppm_ shift)^2^), where N_ppm_ shift is the difference in ppm in the nitrogen dimension and H_ppm_ is the difference in ppm in the proton dimension. The perpendicular distance (p) between the resonance and the CCSP line was calculated in the following way: p = √(x^2^−s^2^), where x = length of line connecting resonance to peptide free resonance and s = scalar projection.

The CCSP value (expressed as a percentage) for each individual resonance was calculated by: CCSP = 100×(scalar projection/CCSP line). The 3 average CCSP values for SI, SII and SII_RT_ were calculated by taking the average CCSP values for the amides from the 3 groups of residues defined in the cluster analysis (see Cluster analysis section) with errors of ±95% confidence intervals.

The combined NH error for any given resonance in an HSQC spectrum was calculated as ±0.0051 ppm (2.5 Hz). This was calculated by taking the average 95% confidence interval value for the mean resonance positions of 4 resonances that showed the smallest differences amongst the 24 complexes. These resonances are from the backbone amide of residues 45, 41 and 27 and the side-chain amide of the C-terminal residue 59 in all complexes (see Fig. 1 and S3, S4, S5, S6). The error for an individual CCSP value is propagated using the combined NH error of ±0.0051 ppm.

### Screening amide groups that were significantly contaminated by the direct effects of peptide sequence differences

**Figure 3:**
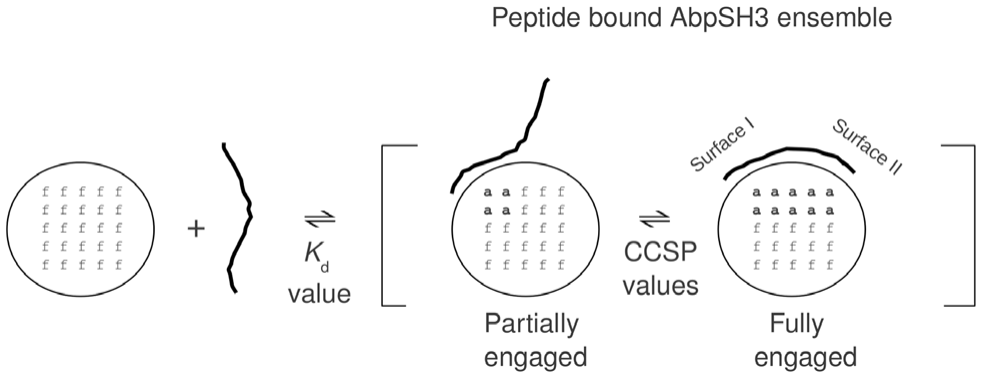
Dynamic exchange model for peptide bound conformers that is consistent with the CCSP phenomena. AbpSH3 is represented by a circle with 25 residues that either exist in the *f* or *a* residue-level conformer. When peptide (drawn as a line) binds and saturates the domain (indicated by the bracketed region), as dictated by its dissociation constant *K*_d_, different peptide bound AbpSH3 conformers can exist in dynamic equilibrium, as dictated by the residue CCSP values. The relative population of each protein conformer is likely influenced by the local binding energy of each region within the particular peptide that binds. It should be noted that an idealized protein conformer with all relevant residues either completely in the *f* or all relevant residues completely in the *a* conformer likely never exists on its own due to the inherent dynamical nature of proteins and as such the peptide-free and fully engaged AbpSH3 conformers modeled by the peptide-free AbpSH3 and the ArkA-AbpSH3 complex in this figure can only be approximations.

For many residue sets, individual resonances within its set did not always fall close to the CCSP line (drawn between peptide free and ArkA bound AbpSH3) as predicted from our model in Fig. 3. The principle reason is due to sequence differences in the peptides masking the expected CCSP behavior in residues that come into direct contact with the peptide (Fig. S2). Thus, to obtain an accurate set of CCSP values the NH resonance sets were carefully analyzed to exclude these contaminated residues and only certain resonances were selected for analysis (Fig. S3, S4, S5, S6 and Table S1). If the chemical shift difference between a given residue's resonance in the peptide free and ArkA-bound AbpSH3 spectrum was less than 0.03 ppm, we considered the chemical shift change to be too small to accurately examine (see 59 s in Fig. S6 as an example), which was the case for 22 residues in the 59 amino acid domain. We evaluated the remaining sets by looking at how many resonances in any given set are close to the CCSP line judged by the length of the perpendicular distance between each resonance to the CCSP line (black line in Fig. 2C). If at least 60% of the resonances in any given set have perpendicular distances that are less than 10% of its CCSP line they were selected for further analysis (Table S1). By examining the internal consistency of each set we provide additional evidence that the location of an amide in the complex is less likely to be contaminated. 15 of the 59 backbone NH groups (and an additional 3 of the 5 side-chain NH groups), involving more than a quarter of the SH3 domain residues, were selected and their CCSP values calculated (Table S1). As can be seen in Table S1, most of the rejected residues are part of SI or II with NH chemical shift changes less than 0.1 ppm and are likely to be significantly affected by contamination from direct sequence differences in the peptides.

It should be noted that the initial analysis of 6 complexes in Fig. 1 involved peptides with the greatest differences in sequence, thus more contamination and a smaller number of residues showing CCSP behavior. On the other hand, the subsequent analysis of the 24 complexes include many more ArkA point mutants and with a bigger dataset allowed detection of CCSP behavior for a larger number of residues using the procedure outlined above.

### Cluster analysis

All CCSP values that met the previously described criteria (See Table S2) were analyzed in the program Cluster 3.0 (using uncentered correlation and average linkage settings) and viewed in the program Java TreeView 1.1.5r2 [29], [30]. Based on the clustering analysis, the average CCSP value of SI uses backbone NH CCSP values from residues 9, 11 and 25; the average CCSP value for SII uses backbone NH CCSP values from residues 30–32, 34, 37–39, 49, 57 and the side-chain NH CCSP value from residue 36; the average CCSP value of the RT loop uses backbone NH CCSP values from residues 13, 15 and 16.

### Estimation of cell growth levels

Using data from [8], we assigned a rating of no growth (value of 0), partial growth (value of 3), intermediate growth (value of 6) or full growth (value of 8), by examining the growth of the cells plated at 37°C. The data for Srv2 were collected at the same time as the others but never published (Bianca Garcia, personnel communication).

### Accession numbers

The assignments of the 24 complexes and the peptide free AbpSH3 have been deposited into BioMagResBank with accession numbers **18054** to **18078**.

## Results

### Co-linear chemical shift perturbations of AbpSH3 target peptide complexes provide evidence for substantial dynamics

We have previously investigated the interaction of the AbpSH3 with six different target peptides derived from yeast proteins, having varying binding affinities (Table S4 and Fig. 1) [8]. At least three of these (Ark1p, Sjl2p, and Scp1p) are proven biological targets of the domain [23]. Ark1p contains two tandem proline-rich binding sites (referred to as ArkA and ArkB) and we have performed experiments on both of these. It should be noted that the sequence of the peptide targets are numbered from N- to C-terminal direction: 6, 5,…, −9,−10, where residues 3 to −3 encompassing the PxxP motif are part of the SI-binding region (Fig. 2) and residues −3 to −8 are part of the SII binding region (shaded grey in sequence alignment in Fig. 2). Comparison of the NH correlation spectrum of the peptide-free AbpSH3 with spectra of it in complexes with six peptide targets revealed significant changes in chemical shifts of residues at SI (residues 8–10, 51, 53, 54) and SII (residues 13–17, 32, 33, 35, 36, 49) and of residues associated with these surfaces (Fig. 1) [8]. Residues associated with SI are found on the first strand and at the end of the RT- loop (colored orange in Fig. 1). Another group of residues, associated with SII, is found in the hydrophobic core, centered on the side-chain of W37 and including E30, F31, D34, L38, G39, F50, S52 and L57 (colored yellow). ^15^N backbone amide order parameters determined for the majority of these residues show that overall they become more rigid upon ArkA target binding (Fig. S7). Note that several residues associated with SI and SII are not close to the binding surface (Fig. 1), for example, for L49/F31 and D33/L57, the closest distances are 8.5 Å and 9.2 Å respectively. Thus, chemical shift and dynamic changes observed for these residues demonstrate that peptide binding can induce longer-range conformational changes in the protein.

Initially we performed relaxation dispersion and R1ρ measurements for several of these AbpSH3 complexes and did not detect chemical exchange for the majority of backbone amides (unpublished data, Stollar EJ and Lundström P), suggesting that any fast exchange dynamics was outside the window of sensitivity for these experiments. We thus turned to CCSP analysis which required much less protein and a fraction of the NMR time and proved to be a rapid and efficient way to explore fast conformational exchange within these complexes. The NH correlation spectra for these 6 studied peptide complexes were overlayed with the peptide-free AbpSH3, and nine residues located in and around SII were found to display clear CCSP behavior with any given residue exchanging between two conformers (Fig. 2). For these residues, the chemical shifts of the amide group in the peptide-free AbpSH3 is at the start of the “CCSP line”, representing the first residue-level conformer, and the position of the amide chemical shifts in the other complexes lie along the line, with the peak furthest along the line, usually from the AbpSH3-ArkA complex, representing the second residue-level conformer. This observation indicates that each of these nine residues undergoes fast exchange on the NMR timescale between these 2 conformers, which we call the *f* and *a* (residue-level) conformers that are based on the chemical shifts of the amides found in 2 AbpSH3 conformers, the peptide-free state and the ArkA-bound state, respectively. In order to quantitate CCSP values, we calculate the projected position of any given resonance along the CCSP line (Fig. 2) and express these values as a percentage, where 0% is the position of the resonance in the *f* conformer and 100% is the position of the resonance in the *a* conformer. It is very important to note that in all these NMR studies on AbpSH3 complexes, we fully saturated AbpSH3 binding (>99%) based on observation of negligible chemical shift changes upon addition of increased amounts of target peptide and/or adding sufficient peptide based on recorded *K*_d_ values (Fig. S1). This measure ensured that observed variations in chemical shift were not simply due to varying degrees of peptide occupancy with exchange occurring between the free and bound states.

Thus, since the nine residues displaying CCSP behavior are found near SII, these results demonstrate that when certain peptides are completely bound to AbpSH3, differential rapid exchange between the *f* and *a* conformers occurs for residues around SII. This must reflect exchange between at least 2 peptide-bound conformers of the SH3 domain, one more similar to the peptide-free state and another similar to ArkA-bound state. Three of the nine residues that display CCSP behavior are binding surface residues (V32, L49 and D34) and had chemical shift values that were more scattered about the line than the others (see Fig. 2). These observations can be explained by the different peptide sequences bound to the SH3 domain contributing to sequence-specific “contamination” of surface residue chemical shift values (such as by changes in local electric fields), adding noise or completely masking the CCSP phenomena. Further measurements with peptide point mutant complexes clearly illustrate the contamination issue, as AbpSH3 residues close to the peptide mutation site in the P(2) series (SI) did not show CCSP behavior although these same SI residues did show this behavior in peptide complexes when the mutation site was further away in the K(−2) and L(−7) series, revealing that as many as 25 out of 59 backbone residues display CCSP behavior within this data set (Fig. S2). Thus, our results show that the different peptide sequences do not directly contribute to the co-linear chemical shift changes via local changes in electric fields (which lead to contamination effects) but indirectly by shifting the relative populations of the 2 peptide-bound conformers of the AbpSH3 domain.

### Evidence for peptide dynamics within the bound state

**Figure 4:**
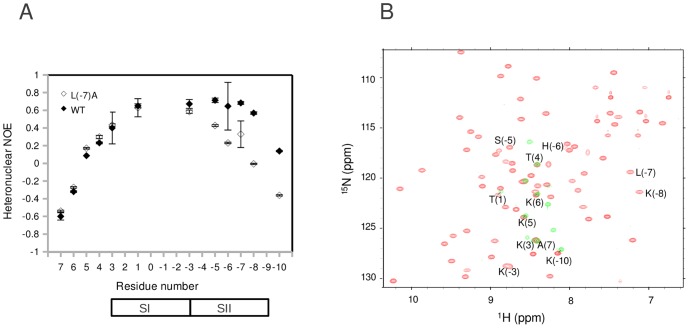
Analysis of peptide dynamics in WT and mutant peptide complexes. (A) Analysis of L(−7)A peptide complex reveals a partially bound and dynamic peptide. ^15^N-^1^H NOE values for AbpSH3-bound ArkA (filled diamonds) and AbpSH3-bound L(−7)A (empty diamonds) show that there is significant dynamics in the C-terminal part of the peptide for the L(−7)A complex. The bars at the bottom of the graph indicate which positions of the peptide bind SI or SII. (B) 2D NH correlation spectrum of AbpSH3-ArkA complex (both protein and peptide in red) and AbpSH3-ArkA L(−7)A complex (only peptide is shown in green). The ArkA peptide resonances are labeled. The N-terminal half of ArkA L(−7)A peptide has NH chemical shift values close to WT (see positions 6, 5, 4, 3 and 1 and the lines that connect the resonances), while the C-terminal half has NH chemical shift values different to WT (see positions −3, −5, −6, −7 and −8) and several are clustered in the center of the spectrum.

Based on the arguments above, our observation of fast exchange at the residue-level indicates that any given complex contains exchanging protein conformers. A simple model to account for this observation is shown in Fig. 3 which depicts peptide bound AbpSH3 in two different conformers that are in rapid exchange in which protein residues in and around the binding interface sample either the *f* or *a* residue-level conformer. As we have no CCSP data on the peptide residues (peptides were unlabeled), in order to explore the behavior of the peptide in AbpSH3 complexes and provide complementary evidence of this dynamic exchange, we compared the backbone dynamics for two ^15^N labeled peptides bound to AbpSH3. The first ^15^N labeled peptide was the ArkA peptide in complex with AbpSH3, having chemical shifts for perturbed AbpSH3 residues lying at the end of the CCSP line on average; the second was an ArkA L(−7)A mutant peptide in an AbpSH3 complex with chemical shifts for perturbed residues lying ∼60% along the CCSP line on average. We measured ^15^N-^1^H heteronuclear NOEs (Fig. 4), which reflect fast timescale (ns-ps) dynamics, on the peptides within both complexes. It is shown that the ArkA peptide is well-ordered throughout its SI- and SII-binding regions and only displays significant flexibility at the peripheries. The L(−7)A peptide shows similar dynamics to the wild-type peptide at the N-terminus and in the SI-binding region but has significantly increased mobility for its SII-binding region, which is the site of the mutation. Comparison of the chemical shift values of the wild-type and L(−7)A peptides shows that the N-terminus and SI-binding region of the peptide have nearly identical values while the chemical shift values of the SII-binding region are very different (Fig. 4B). These chemical shifts of the L(−7)A peptide are closer to the central region of the spectrum (∼8.2 pm), consistent with its SII-binding region within the AbpSH3 complex being more disordered. These results demonstrate that rapidly exchanging protein conformers can be created when a peptide (that is >99% bound to AbpSH3) is not completely engaged with the binding surface due to some parts of the peptide (in this case the SII-binding portion) interacting more weakly than others with fluctuating contacts to the domain, reminiscent of short α-helical peptides that fray at their ends and populate a range of states [31].

### CCSP values vary between peptide complexes

In addition to the 6 target peptide complexes and the 6 complexes within the ArkA mutant series described above, we examined the chemical shift positions for 12 other peptides to create an NH CCSP value database from 24 AbpSH3 complexes and the peptide-free AbpSH3, as described earlier (see Fig. 2). This set of peptides included ArkB, additional ArkA mutants and variations on the length of a number of the peptides including a short 10-residue ArkA peptide (residues 6 to −3) that primarily binds SI (Table S3). We also recorded ^1^H-^13^C constant time HSQC spectra for the K(−3) series and found several side chain CH groups also displaying CCSP behavior (Figs. S2, S8) that augment this database. For our analysis using this large collection of data, the NH resonance position from each of the 24 peptide complexes and that from the peptide-free AbpSH3 were superimposed for each AbpSH3 residue in turn (Fig. S3, S4, S5, S6) and carefully analyzed. 15 backbone NH groups and an additional 3 of the 5 side-chain NH groups, involving more than a quarter of the SH3 domain residues, were found to have large enough chemical shift changes and sufficiently free from contaminating effects to be analyzed further (see Materials and Methods for details).

**Figure 5:**
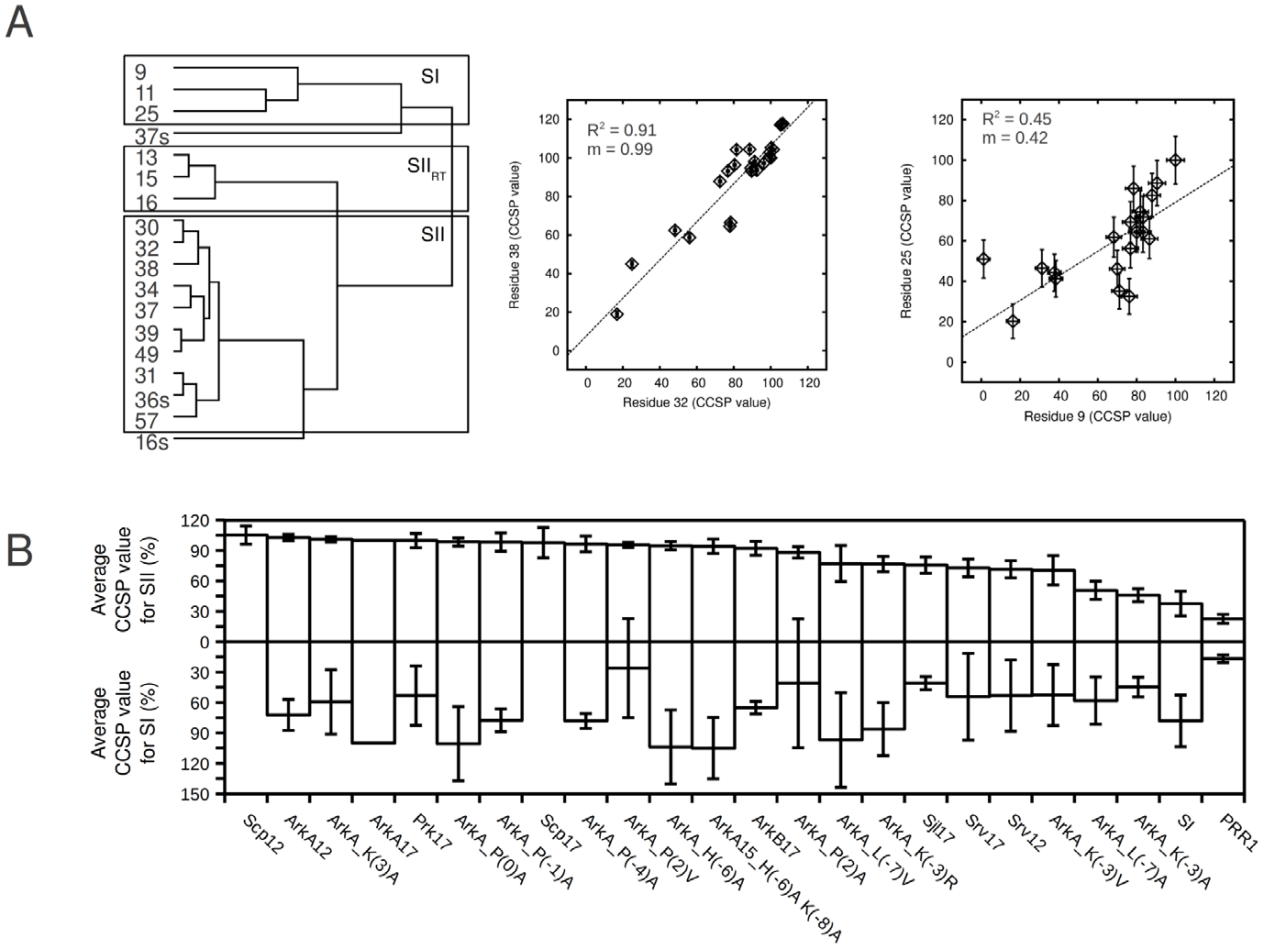
CCSP analysis of 24 AbpSH3-peptide complexes. (A) Left. CCSP values from 24 different peptide complexes are used in multiple pairwise comparisons to group correlated residues into a dendogram where the shorter the line connecting items indicates a closer relationship. The dendogram is grouped in three sections indicating correlated groups of residues for SI (top box), the SII portion of the RT loop tip (middle box) and SII (bottom box) that are used in this study. The side-chain NH groups of residues are denoted by the residue number followed by an “s”. The programs Cluster 3.0 and Java TreeView 1.1.5r2, were used for the clustering analysis and tree visualization, respectively. Right. 2 pairwise correlations of CCSP data (V32 *vs.* L38 and D9 *vs.* K25). (B) Average CCSP values for SI and SII. Bars indicate ±95% confidence intervals for the average and are substantially larger for SI, due to chemical shift changes occurring amongst a fewer number of residues and with smaller magnitudes in this region. Missing average values indicate residues contributing to the average were considered contaminated by our selection procedure.

In order to identify which residues within AbpSH3 populate the *a* conformer to the same extent and are therefore potentially coupled within the domain∶peptide ensembles we used a clustering algorithm [29], [30] which clusters SH3 domain residues based on their CCSP values, as has been used in another NMR chemical shift study as well as gene array studies using microarray data [21], [32]. With our set of CCSP values (Table S2), pairwise comparisons across all residues in all peptide complexes were used by the algorithim to establish a hierarchical tree, with branch lengths indicating how similar any two items are to each other (Fig. 5A). As can be seen in Figure 5A, the majority of residues were grouped as would be expected based on structural proximity within AbpSH3. The residues in the top box are connected to surface I, the residues in the middle box are connected to the tip of the RT loop which is part of surface II. The residues in the bottom box are also part of surface II or nearby. Within the last group, the algorithm finds close correlations between 30, 32 and 38, which have amide groups close together and pointing towards each other in the structure. It also correlates residues 34 and 37 which have amides 6 Å apart as well as 39 and 49 which have amides 4.6 Å apart on adjacent beta strands. It is also clear from Fig. 5A,B that the correlations between residues within the SI group are much poorer than SII leading to larger confidence intervals for the average. This is due to the fact that less conformational change appears to take place in SI, leading to fewer residues representing this region and with much smaller chemical shift changes (on average 4 times smaller shifts than SII with half as many residues, see Table S1) and a lower average order parameter change (0.010 for SI vs. 0.025 for SII, see Fig. S7). However, the fact that there is a range of CCSP values in SI suggests that an additional partially engaged conformer must exist where SI is not fully engaged (Fig. S12A). The only residues that were not correlated strongly with other residues (besides to their own backbone amide) are the side-chain amides of W37 and N16. Based on this clustering analysis, we chose to average the CCSP values of each group to calculate mean conformer exchange at SI, SII and the RT-loop. As expected from the clustering analysis there is a weaker correlation between these average values for the SI and SII surfaces (Fig. S9, R^2^ = 0.35), while there is a better correlation between SII and the RT-loop (Fig. S9, R^2^ = 0.59).

To further validate our clustering analysis approach and explore the coupling between residues in all groups, we made pairwise plots of CCSP values from several pairs of residues. We found expected correlations between NH groups within the same clustered group as well as between side-chain and backbone NH groups from the same residue (Fig. 5A, S9). We also found much poorer correlations between residues from SI and SII (Fig. S9).

Fig. 5B and Table S4 show, for all 24 complexes, the average population of the *a* conformer for both SI and SII backbone NH groups (according to groupings in Fig. 5A). Values range from ∼20 to 100% for both SI and II, reflecting the different populations of protein conformers for each protein-peptide complex (recall again that each complex is >99% peptide-bound). This range reflects the sequence differences within the peptides that must lead to different local binding energies along the peptide chain leading to different extents of AbpSH3 engagement and thus different populations of the bound state conformers (Fig. 3).

### Thermodynamic and structural signatures of dynamic exchange

**Figure 6:**
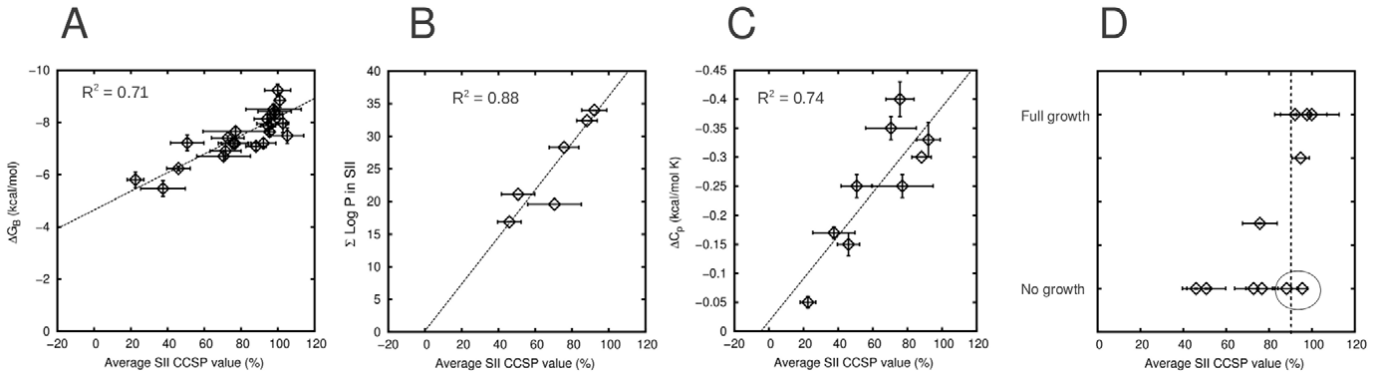
Correlations of thermodynamic and CCSP data. (A) Correlation between global peptide binding energy, ΔG_B_, and the average CCSP value for SII for all 24 complexes. (B) Average CCSP value for SII *vs.* the sum of log (backbone amide protection factors) in SII as measured in H-D exchange experiments (ΣLogP in SII). (C) Average CCSP value for SII *vs.* ΔC_p_. (D) Average CCSP value for SII *vs.* biological growth. The dotted line indicates the possible threshold CCSP value that determines cell growth. The circled data points are the P(−2)V and P(−2)A complexes which would be expected to have better growth based on their average SII CCSP value (discussed in text). Errors are ±95% confidence intervals except the ΔC_p_ values which report the standard error of the least squared fit to the data (ΔH *vs.* temperature).

As can be seen in Figure 3, the global binding energy change (ΔG_B_) for complex formation is related to the equilibrium populations of the peptide-bound and free SH3 domain conformers, in contrast to the CCSP value, which reflects the equilibrium populations of 2 residue-level conformers within the peptide-bound dynamic ensemble (when measured in peptide saturating conditions). In order to explore the link between local conformational sampling and global thermodynamics of peptide binding, we measured ΔG_B_ values for 8 AbpSH3-peptide complexes (Table S4) using isothermal titration calorimetry (ITC), augmenting the data previously obtained [8], to generate a complete data set for the 24 complexes for which we have CCSP data. Additional measurements reveal that the 8-residue SII-binding region peptide (residues −3 to −10) gives no detectable binding by ITC or NMR titrations (data not shown). The correlation between ΔG_B_ and the average CCSP value for SI was poor with an R^2^ = 0.33 (Fig. S9A) which may be due to the large CCSP errors in SI. A good correlation was observed with the average CCSP value for SII giving an R^2^ = 0.71 (Fig. 6A) suggesting that many peptides differ in binding energy due to differences in binding at SII and likely have fairly similar binding energies for SI.

In order to explore other correlations with the biophysical properties of the complexes, we measured hydrogen exchange protection factors for a variety of peptide-saturated complexes and compared them to our CCSP data. We expected that protection would be highest for domain∶peptide complex ensembles that populate the *a* conformer for most of its residues (high average CCSP values) based on the physical protection and overall reduction in dynamics based on NMR relaxation data (Fig. S7). As expected, the log of hydrogen exchange protection factors increased throughout AbpSH3 in all complexes, relative to the peptide-free state, and with a net increase in protection in SI and SII (Fig. S10). When we compared the sum of the log of protection factors for SII residues (that showed quantifiable changes; residues 13–17, 32–33, 35–36 and 49, predominantly in loops) for all tested complexes, we found a good correlation with the average CCSP value for SII (R^2^ = 0.88 see Fig. 6B) which improved further if we included the RT-loop residues 12, 13 and 16 (R^2^ = 0.98) with an extrapolation to almost zero protection when the average CCSP value for SII is 0.

To obtain further thermodynamic insights into binding, we measured the change in heat capacity (ΔC_p_) for several complexes using ITC. We found a good correlation between ΔC_p_ and the average CCSP value for SII for these complexes (R^2^ = 0.74, see Fig. 6C) and, in contrast, a poorer correlation with SI (Fig. S9B, R^2^ = 0.04). This is in agreement with other thermodynamic studies [33] showing that the greater the amount of hydrophobic burial and restrictions in soft motional modes (predominantly in and around SII), the more negative the heat capacity change becomes [34], [35]. AbpSH3:ArkA17 complex formation leads to a ΔC_p_ of −0.36 kcal/mol K which may be accounted for by some combination of the burial of SII, the folding and burial of the C-terminal part of the peptide (all leading to hydrophobic burial) and conformational restrictions in and around SII. As less SII is engaged in the other complexes, ΔC_p_ becomes more positive, with an extrapolation to 0 ΔC_p_ when the average CCSP value for SII is 0. This likely reflects a less specific dynamic complex where the surface is loosely bound, similar to non-specific loosely bound protein:DNA complexes that also have negligible ΔC_p_ values [36]. These observations suggest that most complexes differ in the extent to which residues in SII are in the *a* conformer and that this reflects the extent the interface is physically protected by peptide binding and/or stabilized by peptide-induced *f*-to-*a* conformational transitions.

### Full peptide engagement is important for AbpSH3 function

The biological importance of the extended peptide binding interface of AbpSH3 has been previously demonstrated using an *in vivo* growth assay with a variety of the peptides studied here. Thus, in order to test the functional relevance of peptide engagement to the specific surface II of AbpSH3 as measured by the average CCSP value for SII, we compared these values to *in vivo* yeast cell growth assay data for 12 peptide sequences [8]. We observe that these average CCSP values appear to define a threshold for the cell growth for each peptide sequence in the assay (Fig. 6D); peptides with an average CCSP value in SII lower than ∼90% lead to no cell growth, peptides with a CCSP value in SII greater than ∼90% lead to robust cell growth and peptides close to this value are in a transition zone for cell growth. P(2)V and P(2)A (Fig. 6D, circled points) have an average CCSP values that would predict strong growth but does not support growth. P(2)A and P(2)V yield almost no reliable CCSP behavior in surface I (or nearby) residues (Fig. S2) making it hard to assess the extent of engagement of SI by NMR. None-the-less, it is reasonable to expect that mutating P(2), which is a core conserved proline in the canonical PxxP motif, will affect peptide engagement of SI, explaining why both mutants have reduced binding energies, are unable to grow well in the assay and cannot be fully predicted by their average CCSP values in SII. A similar correlation is seen between binding affinity and growth [8], yet the physical model underlying CCSP values pointing to the different local binding at SI and SII between the complexes may enable future experiments to give additional insight into the sub-surface thresholds required for growth. Thus, for a functional interaction, our data indicates that the engagement of both surfaces of AbpSH3 is important, making CCSP measurements useful for predicting and understanding functional properties of different biological complexes.

## Discussion

The chemical shift and relaxation rate changes that occur when AbpSH3 binds peptide demonstrate both surface and longer-range effects of the interaction. In particular, the analysis of the chemical shift data in the form of co-linear chemical shift perturbation (CCSP) values for both backbone and side chain positions provides evidence that any given peptide-bound AbpSH3 is rapidly inter-converting between at least 2 peptide-bound conformers resulting in different proportions of the *f* and *a* conformers for many of its residues; this work complements and extends other studies using CCSP analysis [12]–[21]. In most studies involving CCSP analysis, conformational equilibria were examined within a single protein (usually containing disordered auto-inhibitory segments). Similar to our work however, 3 studies have used CCSP analysis to understand biomolecular protein interactions, dynamics and function [20]–[22]. Protein Kinase A catalytic subunit (PKA-C) was shown to progress between two major conformations as peptide ligand, ATP and magnesium ions bound within 4 different binary and tertiary complexes [22]. An amphipathic helix from phospholambin was shown to exchange between a structured and disordered form and, using 7 different helix peptide variants, the population of the disordered form was found to correlate with its phosphorylation efficiency by PKA-C [20]. Like these studies, our work also points to the need to populate a specific conformation (that of the SH3 domain fully engaged at SII) in order to achieve a functional outcome and that a range of ensembles can exist depending on which local protein interactions are present. Chemical shift perturbations were also shown to occur in saturated complexes of the EPAC cAMP binding domain with a variety of small molecule ligands, facilitating covariance analysis to dissect the effects of direct binding and allostery [21]. This study and ours highlight the wealth of information that can be extracted from CCSP analysis of 2D spectra from a group of related proteins. Our work additionally provides new insights into the behavior of extended target peptides for interaction domains, suggesting that various regions of the peptide can behave differently and semi-independently with functional consequences as discussed below.

Particular peptide-bound conformers are likely be populated in all the peptide complexes we have characterized. This includes the functionally important “fully-engaged state” that is modeled by the ArkA:AbpSH3 complex as well as some partially engaged conformers where peptide is fully engaged to only one surface. We find that the fully engaged conformer (peptide bound conformer on the right in Fig. 3) is more restricted/protected than the other peptide bound conformers in the ensemble. This is supported by its more favorable global binding energy leading to the most negative ΔC_p_, the most SII hydrogen exchange protection with more favorable enthalpy and a reduction in favorable entropy as well as reduced backbone dynamics compared to the peptide-free conformer. The peptides that give the lowest CCSP values in AbpSH3 are the PRR and SI-binding peptides, both of which have the SI binding proline rich region but completely lack the sequence necessary to bind surface II. These peptides clearly bind exclusively to SI (peptide bound conformer on the left in Fig. 3) and approximate a partially engaged conformer. These peptides cause minimal conformational change across the domain, their ΔC_p_ value and SII hydrogen exchange protection are close to 0 and their binding energies are the lowest measured. The 2 types of complexes discussed above, may represent some of the major peptide bound conformers present in the bound-state ensemble and their future study will provide deeper insights into SH3 domain-peptide binding pathways.

Another partially engaged conformer which may be highly populated in certain peptide complexes and is not depicted in Fig. 3 is where peptide partially engages SI (Fig. S12A). For example, as expected, CCSP values for the P(2) mutants suggest that SII is bound more than SI which must come from fast exchange with this type of conformer. A partially engaged SI in these mutants is also consistent with the yeast cell growth data and suggests that although SI is common to most SH3 domains and may not provide opportunities for specificity enhancement, it may play a role in binding affinity/kinetic enhancement. In fact, unlike most other studies to date, closer analysis of CCSP values within any given complex reveals significant residue-to-residue variability (Fig. S11A) which suggests that the exchange depicted in Fig. 3 may be more complicated and may comprise other partially engaged conformers. Assuming contamination effects along the CCSP line are negligible (especially for amide groups further from the binding surface), from a simple mathematical standpoint, if only two AbpSH3 peptide bound conformers existed, only 2 possible groups of CCSP values would exist amongst the AbpSH3 residues (Fig. S11B). However, we find that residues show a range of significantly different values; for example, within the SII group for complexes in Fig. S11A, residues from each boxed region have non-overlapping confidence intervals. This indicates at least 3 significantly different CCSP values within SII and its surrounding area, suggesting greater than 2 state exchange at the protein level (Fig. S11C and S12).

As in one other chemical shift perturbation study investigating protein interactions [21], binding cooperativity between different binding sites via conformational coupling or allostery [1], [37] can be examined. The model depicted in Fig. 3 suggests that changes in local peptide binding to SII cause local and independent *f*-to-*a* residue-level conformer changes in AbpSH3. However, an alternative model whereby changes in local peptide binding can cause changes in *f* and *a* conformer exchange at some other distant (binding) site in the domain is possible (Fig. S12B). In support of this, Fig. 4 shows that even though peptide dynamics and chemical shift values (reflecting binding) were almost identical between complexed L(−7)A and wild-type peptides in the SI region, a much greater population of *f* conformers is seen in SI for the L(−7)A peptide complex, suggesting conformational coupling between SI and SII. Further evidence for conformational coupling between SI and SII comes from studies with the 10-residue primarily SI-binding peptide (residues 6 to −3) from ArkA. When this peptide is bound to AbpSH3, residues in and around SII transition to the *a* conformer, with chemical shift changes of nearly a third to a half of those in the ArkA peptide complex (Fig. S8, Table S4).

An alternative explanation for the CCSP phenomena is that every peptide sequence causes specific chemical shift changes due to the differing chemical environment they present to the domain residues and that there is one common AbpSH3 conformation for all exchanging bound-state conformers that only differ in the extent the peptide is engaged. However, the point mutant data (Fig. S2) strongly suggests direct contamination effects take place locally and any other chemical shift changes must be explained by exchange between at least 2 AbpSH3 conformations which is supported by the conformational coupling that appears exists between SI and SII. Furthermore, the structural differences seen between the peptide-free AbpSH3 (representing *f* conformers) and the ArkA-bound AbpSH3 (representing *a* conformers) also support this notion. There is an overall backbone RMSD between the free and ArkA bound AbpSH3 structures of 0.84 Å with significant C_α_- C_α_ distances in the N-terminal strand (1.2 Å), C-terminal strand (8.0 Å), the RT loop (3.0 Å) and the n-src loop region (1.3 Å) [8], which are regions that display CCSP behavior and include regions distant to the binding site. Furthermore, 8 of the 18 NH groups that show CCSP behavior are found on flexible loops and a further 4 are found on the less constrained first and last strands. These regions are more likely to show binding induced conformational changes, which is also supported by the relaxation data (Fig. S7). Interestingly, the two structures reveal several potential coupling sites that may explain how changes at SI are coupled to SII, where residues from the SI cluster are close to residues in the SII cluster.

Our work provides general insights into peptide binding, highlighting that any given peptide has two important properties with respect to the peptide∶domain complex (Fig. 3). The first is a well-established property, that of its global binding energy or related dissociation constant, which will dictate the necessary concentrations of domain and peptide to achieve a certain level of peptide occupancy and thus functional binding inside the cell. The second, revealed in this study, is the *extent* a peptide engages the complete binding surface. Every peptide we studied was bound greater than 99% of the time, although the *extent* they engaged AbpSH3 varied dramatically even amongst complexes that had almost identical global binding energies. The structural and functional diversity we measure appears to be due to the fact that peptides (and flexible binding loops) do not have constrained tertiary structure. Instead a peptide can be regarded as containing several semi-independent segments along the chain, whereby at any moment one segment may be bound to part of the surface of the domain while the other is not bound due to differences in their *local* binding energies. Most modular binding domains target sequence motifs located within disordered regions of proteins [38], suggesting that the ensemble model we have presented may be general for many other interactions. Furthermore, analysis of several different protein-protein complex families such as antibody-antigen, TCR-MHC peptide and nuclease-inhibitor complexes has revealed interfaces that can be divided into common and variable surfaces amongst the domain family members, similar to the SH3 domain surfaces I and II [39]–[41]. We predict that when large surfaces (that may contain flexible binding loops) bind an extended disordered peptide target, a dynamic ensemble will be formed due to the differing *local* binding energies within the interface, with the relative populations of each conformer in that ensemble being crucial for function and its biophysical properties.

In all complexes, CCSP behavior occurs when two or more bound conformers at the protein level are in fast exchange. This has significant consequences for NMR structural studies, since without careful analysis of the NMR data it is easy to assume that a protein complex having one set of peaks in a 2D correlation spectrum exists in one stable conformer, when it may be highly dynamic and exchanging between multiple conformers. Such dynamic exchange poses technical challenges for NMR structure determination methods as chemical shifts average differently than NOEs and other structural data. It is tempting to speculate that many previously studied proteins and protein complexes have been selected based on their stability and the presence of one dominating conformer (such as the highly engaged ArkA complex) and that complexes with significant populations of alternative bound conformers (such as Sjl2 or ArkB complexes) are not pursued due to technical problems that may arise during structure determination. However, increasingly challenging targets for structural work are being studied and the possibility of fast exchange between multiple bound conformers must be addressed [42], [43].

This study lays the foundation for modeling ensembles using co-linear chemical shift perturbations as constraints, which promise to provide deeper insights into the physico-chemical basis of biologically relevant protein interactions.

## Supporting Information

Figure S1 **Representative peptide titrations of AbpSH3.** The peptide titrated is indicated above each graph as well as the fitted *K*_d_ value and maximum change in ppm. All titrations follow the NH chemical shift change of F31 except L(−7)V which follows A13, both of which have large chemical shift changes when ArkA binds AbpSH3. The data were fitted to a standard binding equation previously described (4). The protein concentration was fixed to a value between 70 and 100 µM and for each peptide concentration an NH-correlation spectra was collected until negligible change occurred. The combined NH error for any given point was calculated as ±0.0051 ppm (2.5 Hz). All *K*_d_ values measured by ITC closely agree with the values determined by these titrations and further indicates that the maximum chemical shift change for a given residue is not necessarily the same across peptide complexes due to CCSP behavior.
(TIF)

Figure S2 **CCSP data for the P(2), K(−3) and L(−7) peptide series designed to minimize peptide sequence contamination.** In order to minimize “contamination” by direct effects of different target peptide sequences, we re-probed the CCSP behavior of AbpSH3 complexes with ArkA-based peptides that would be hypothesized to display a range of SI or SII binding through point mutations of the three most energetically important ArkA positions, P(2), K(−3) and L(−7) (see highlighted positions in Fig. 2A inset, Table S3 and Table S4). Three different series were generated to minimize sequence specific “contamination”. Superposition of NH correlation spectra for complexes from the three series shows that many additional residues display CCSP behavior (Fig. S2D), including nearly all SI and SII residues and most residues close in space to SII. (A) CCSP behavior is found for 9 AbpSH3 residues in the P(2) series (representing a range of surface I binding [WT ArkA, P(2)A and P(2)V]) as seen in an overlay of 4 HSQC spectra (peak positions are represented by squares for peptide-free, up-triangle for “A” mutant, down triangle for “V” mutant and circle for ArkA-bound). The mutations were made in the SI-binding region of the peptide leading to contamination effects on the SI amides (that have smaller chemical shift changes, Table S1), however, CCSP behavior was seen in SII residues, providing evidence that SI and II are linked via conformational coupling. (B) CCSP behavior is found for 16 AbpSH3 residues in the K(−3) series (probing the boundary of surfaces I and II [WT ArkA, K(−3)A and K(−3)V], including those in both SI and SII). This result is expected as the site of mutation is close to both surfaces. (C) CCSP behavior is found for 15 AbpSH3 residues in the L(−7) series (representing a range of surface II binding [WT ArkA, L(−7)A and L(−7V)]). While the mutations were made in the SII binding region of the peptide, CCSP behavior is seen in SI residues, providing more evidence that SI and SII are linked via conformational coupling. It should be noted that even when the mutations were made close to SII residues minimal contamination effects were seen in SII, this is due to the much larger chemical shift changes that occur for these residues as compared to SI (see Table S1) (D) Summary of residues displaying CCSP behavior. Results of different experiments probing N-H, CA-HA and C-H (side-chain) correlations are tabulated. The residues that are part of the binding surface are in bold on a light grey background for SI and are in white on a dark grey background for SII. The summary includes data from aliphatic correlations in spectra of AbpSH3 and AbpSH3 in complex with ArkA WT, K(−3)V and K(−3)A (see Fig. S8B).
(TIF)

Figure S3 **Overlay of NH correlation spectra of all 24 AbpSH3 complexes for residue resonance sets (5–15) with NH** ***f-*** **to** ***-a*** **shifts of 0.03 ppm or greater.** The identity of the NH group plotted is indicated by the residue number inside the plot. All 24 complexes and the peptide-free AbpSH3 are included in these plots, where the peptide-free AbpSH3 (*f* conformer) is indicated by a filled diamond and the ArkA-bound AbpSH3 (*a* conformer) is indicated by the filled square. The first plot for each set is plotted at the same scale for all residues, while the axes in second plot are chosen to show the data more clearly. The error of a resonance position is calculated as ±0.0051 ppm for H and ±0.051 ppm for N.
(TIF)

Figure S4 **Overlay of NH correlation spectra of all 24 AbpSH3 complexes for residue resonance sets (16–31) with NH** ***f-*** **to** ***-a*** **shifts of 0.03 ppm or greater.** The identity of the NH group plotted is indicated by the residue number inside the plot. All 24 complexes and the peptide-free AbpSH3 are included in these plots, where the peptide-free AbpSH3 (*f* conformer) is indicated by a filled diamond and the ArkA-bound AbpSH3 (*a* conformer) is indicated by the filled square. The first plot for each set is plotted at the same scale for all residues, while the axes in second plot are chosen to show the data more clearly. The error of a resonance position is calculated as ±0.0051 ppm for H and ±0.051 ppm for N.
(TIF)

Figure S5 **Overlay of NH correlation spectra of all 24 AbpSH3 complexes for residue resonance sets (32–52) with NH** ***f-*** **to** ***-a*** **shifts of 0.03 ppm or greater.** The identity of the NH group plotted is indicated by the residue number inside the plot. All 24 complexes and the peptide-free AbpSH3 are included in these plots, where the peptide-free AbpSH3 (*f* conformer) is indicated by a filled diamond and the ArkA-bound AbpSH3 (*a* conformer) is indicated by the filled square. The first plot for each set is plotted at the same scale for all residues, while the axes in second plot are chosen to show the data more clearly. The error of a resonance position is calculated as ±0.0051 ppm for H and ±0.051 ppm for N.
(TIF)

Figure S6 **Overlay of NH correlation spectra of all 24 AbpSH3 complexes for residue resonance sets (53–58 and all side-chain amides) with NH** ***f-*** **to** ***-a*** **shifts of 0.03 ppm or greater.** The identity of the NH group plotted is indicated by the residue number inside the plot. All 24 complexes and the peptide-free AbpSH3 are included in these plots, where the peptide-free AbpSH3 (*f* conformer) is indicated by a filled diamond and the ArkA-bound AbpSH3 (*a* conformer) is indicated by the filled square. The first plot for each set is plotted at the same scale for all residues, while the axes in second plot are chosen to show the data more clearly. The error of a resonance position is calculated as ±0.0051 ppm for H and ±0.051 ppm for N.
(TIF)

Figure S7 **Backbone dynamics of the free and peptide-bound AbpSH3** (A) Order parameters calculated for the peptide-free (filled squares) and ArkA-bound AbpSH3 (open squares). See Protocol S1 for experimental details. (B) Difference plot of order parameters. The largest changes occur in the binding surface with a lesser effect seen in the residues associated with surfaces I and II. An R_ex_ term (which is indicative of ms-µs conformational exchange) could be fit to data for 5–6 resonances in the peptide-free state that include the residues associated with the binding surface suggesting there is an exchange of conformations in the absence of peptide. Overall, the bound state is more rigid than the peptide-free state as can be seen in this difference plot (17 residues increase S^2^, 3 residues decrease S^2^), where the largest statistically significant changes are found for residues 33 and 34 in the N-Src loop which is part of SII. Three residues (30, 49, 52) appear to increase flexibility upon binding, although there are large errors associated with these residues. The R_ex_ term could not be fit to data for any residue in the bound state, although a faster timescale term, t_e_ was fit for residue 14, 15, and 16. Taken together, peptide binding reduces the backbone dynamics in AbpSH3 in both the ns-ps and ms-µs timescale regimes, involving residues in and around surface I and II. As noted here and in our previous studies, peptide binding does not completely dampen the dynamics in the tip of the RT- loop (residues 14, 15, 16).
(TIF)

Figure S8 **Further evidence for protein conformational effects within the dynamic bound-state ensemble of AbpSH3:peptide complexes.** (**A**) CCSP behavior is seen in and around SII for the complex with a 10-residue primarily SI-binding peptide from ArkA. Three examples are shown for residues L38, F31 and N16. All of these residues are far from the SI-binding site, suggesting that SI and SII are connected through conformational coupling. The labels are as follows; square is peptide-free AbpSH3, down-triangle is SI-binding peptide complex and circle is ArkA complex. (**B**) CCSP behavior found for aliphatic C-H correlation peaks in the K(−3) peptide series. Overlay of spectra (peak positions are represented by squares for peptide-free, up-triangle for “A” mutant, down triangle for “V” mutant and circle for ArkA-bound) highlighting CCSP behavior for CA-HA correlations and side-chain C-H correlations. The CCSP values from this analysis confirm P51 undergoes conformational exchange (which could not be determined from previous experiments that only probed amide groups) and revealed a general correlation with their amide groups. It should be noted that significant spectral crowding and overlap of aliphatic resonances made it difficult to identify as many examples of CCSP behavior for CH groups.
(TIF)

Figure S9 **Supporting data for CCSP analysis.** CCSP pairwise correlation plots. m is the slope of the best fit line. The outliers in the SII *vs.* SII_RT_ plot were from ArkA K(−3)A, ArkA K(−3)V, Scp12 and Scp17 suggesting the tip of the RT loop is contaminated in these complexes.
(TIF)

Figure S10 **Correlation of thermodynamic parameters with average CCSP value for SI.** (A) Average CCSP value for SI *vs*. ΔG_B_. (B) Average CCSP value for SI *vs.* **Δ**C_p_. Data are mean ±95% confidence intervals except the ΔC_p_ values which report the standard error of the least squared fit to the data (ΔH *vs.* temperature). (C) Hydrogen exchange protection data for peptide-free and ArkA-bound AbpSH3. Hydrogen exchange protection factors obtained for peptide-free AbpSH3 (diamonds) and ArkA bound AbpSH3 (squares). Protection factor values of 0 indicate residues exchanging at rates that are too fast to measure, and protection factor values of 7 indicate residues exchanging at rates that are too slow to determine accurately or did not undergo observable exchange. Several of the squares and diamonds are overlapping with a value of 0.
(TIF)

Figure S11 **Variability in CCSP values within the complexes.** (A) Individual SII residue CCSP values for 2 ArkA mutant complexes, the error bars are 95% confidence intervals and the boxes reveal at least three residues with statistically different CCSP values (non-overlapping error bars). (B) A possible 2 macro-state model. Two residues are considered (broken circle and square) in an ensemble with only two exchanging protein conformers (*W* and *Z* conformations), which are populated W% and Z%, respectively. In this ensemble the CCSP value for both residues would be Z% as both residues are only in the *a* residue-level conformer in the *Z* protein conformer. (C) An alternative multi-macro-state model. The same two residues are considered in an ensemble with four exchanging protein conformers (*W*, *X*, *Y* and *Z* conformations), which have the population ratio W∶X∶Y∶Z. In this ensemble the CCSP value for the residue highlighted by a square would be (Y+Z)% as it is only in the *a* residue-level conformer in the *Y* and *Z* protein conformer. However the residue highlighted by the circle will have a CCSP value of (X+Y+Z)% because it is in the *a* residue-level conformer in the *X, Y* and *Z* protein conformers. By having more than two protein conformers that have different compositions of *f* and *a*, individual residues throughout the domain are able to show a range of different CCSP values because the combination of protein conformers where one particular residue is in the *a* residue-level conformer is likely to be different to the combination of protein conformers where another residue is in the *a* residue-level conformers. The behavior of the peptide that is presented in this model is only one of many possible scenarios (see Fig. S12).
(TIF)

Figure S12 **Possible models for the AbpSH3:peptide ensemble.** (A) Protein residues undergo *f*-to-*a* exchange as the physical association of the peptide increases then decreases across the binding surface. (B) Protein residues undergo *f*-to-*a* exchange as a conformational response to partial binding of the peptide to part of the binding surface. (C) Protein residues undergo *f*-to-*a* exchange as peptide that is physically associated to the complete binding surface becomes more tightly engaged then less tightly engaged, for example by progressively expelling/incorporating waters or counter ions at the interface or refinement of side-chain packing at the interface. The actual ensemble may also be any combination of these models.
(TIF)

Table S1 **Summary of data used to screen amide groups that were significantly contaminated by the direct effects of peptide sequence differences.** Δδ is the combined chemical shift difference between the resonance of the amide in the peptide-free AbpSH3 and the corresponding resonance in the ArkA-bound AbpSH3. # close to the line refers to the number of resonances in any given residue set that has perpendicular distances that are less than 10% of its CCSP line. Those residues that have a δΔ of 0.03 ppm or greater and belong to a set that has 60% or greater perpendicular distances that are less than 10% of its CCSP line were deemed most likely free of contamination and chosen for further analysis (indicated by a star in the fourth column). Residue numbers followed by “s” refer to the side-chain NH group. SI residues are highlighted in red and SII residues in blue. The small table below the main table, indicates the number of residues that have a Δδ of 0.03 ppm or greater in each surface, as well as its average value. SI data is much more limited in this type of analysis, due to fewer residues involved in chemical shift changes and with much smaller magnitude compared to SII.
(TIF)

Table S2 **CCSP values for all residues analyzed further in this study.** As mentioned in Table S1, several SI-associated residue values are missing due to the fact that chemical shift changes were much smaller in this region compared to SII which allows for greater contamination effects. Residue numbers followed by “s” refer to the side-chain NH group.
(TIF)

Table S3 **Alignment of sequences used in this study.** Residues in bold are mutation sites. The sequences are ordered according to their average CCSP values in SII.
(TIF)

Table S4 **Parameters for binding of the 24 studied peptides to the AbpSH3.** The sequences are ordered according to their average SII CCSP value. Binding data for peptides not in bold were reported in (*8*). Errors are ±95% confidence intervals except ΔC_P_ values which report the standard error of the least squared fit to the data (ΔH *vs.* temperature). PRR1 is a 15-residue peptide found N-terminal to AbpSH3 (residues 516 to 530 in Abp1p). SI is a 10-residue primarily surface I-binding peptide (residues 6 to −3) from ArkA. Peptide sequences can be found in Table S3.
(TIF)

Protocol S1 (DOCX)
