# Supplementary figures and images for: Differential Dynamic Engagement within 24 SH3 Domain: Peptide Complexes Revealed by Co-Linear Chemical Shift Perturbation Analysis
Source: PLoS One. 2012 Dec 12;7(12):e51282. doi: 10.1371/journal.pone.0051282 (PMC3520974; doi:10.1371/journal.pone.0051282)

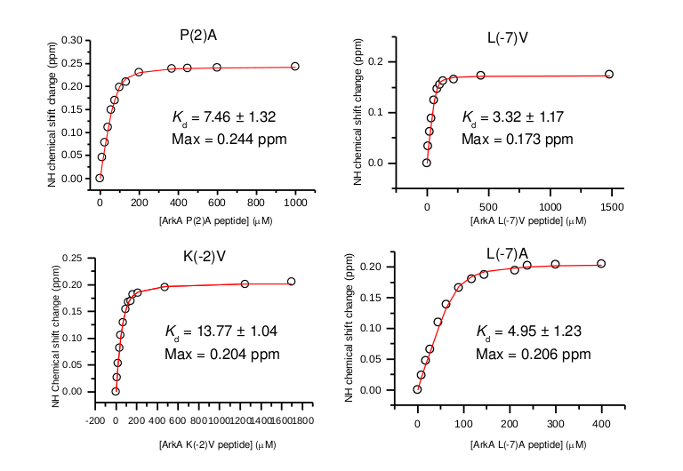

Supplement: Figure S1 — Representative peptide titrations of AbpSH3. The peptide titrated is indicated above each graph as well as the fitted K d value and maximum change in ppm. All titrations follow the NH chemical shift change of F31 except L(−7)V which follows A13, both of which have large chemical shift changes when ArkA binds AbpSH3. The data were fitted to a standard binding equation previously described (4). The protein concentration was fixed to a value between 70 and 100 µM and for each peptide concentration an NH-correlation spectra was collected until negligible change occurred. The combined NH error for any given point was calculated as ±0.0051 ppm (2.5 Hz). All K d values measured by ITC closely agree with the values determined by these titrations and further indicates that the maximum chemical shift change for a given residue is not necessarily the same across peptide complexes due to CCSP behavior. (TIF) [file pone.0051282.s001.tif]

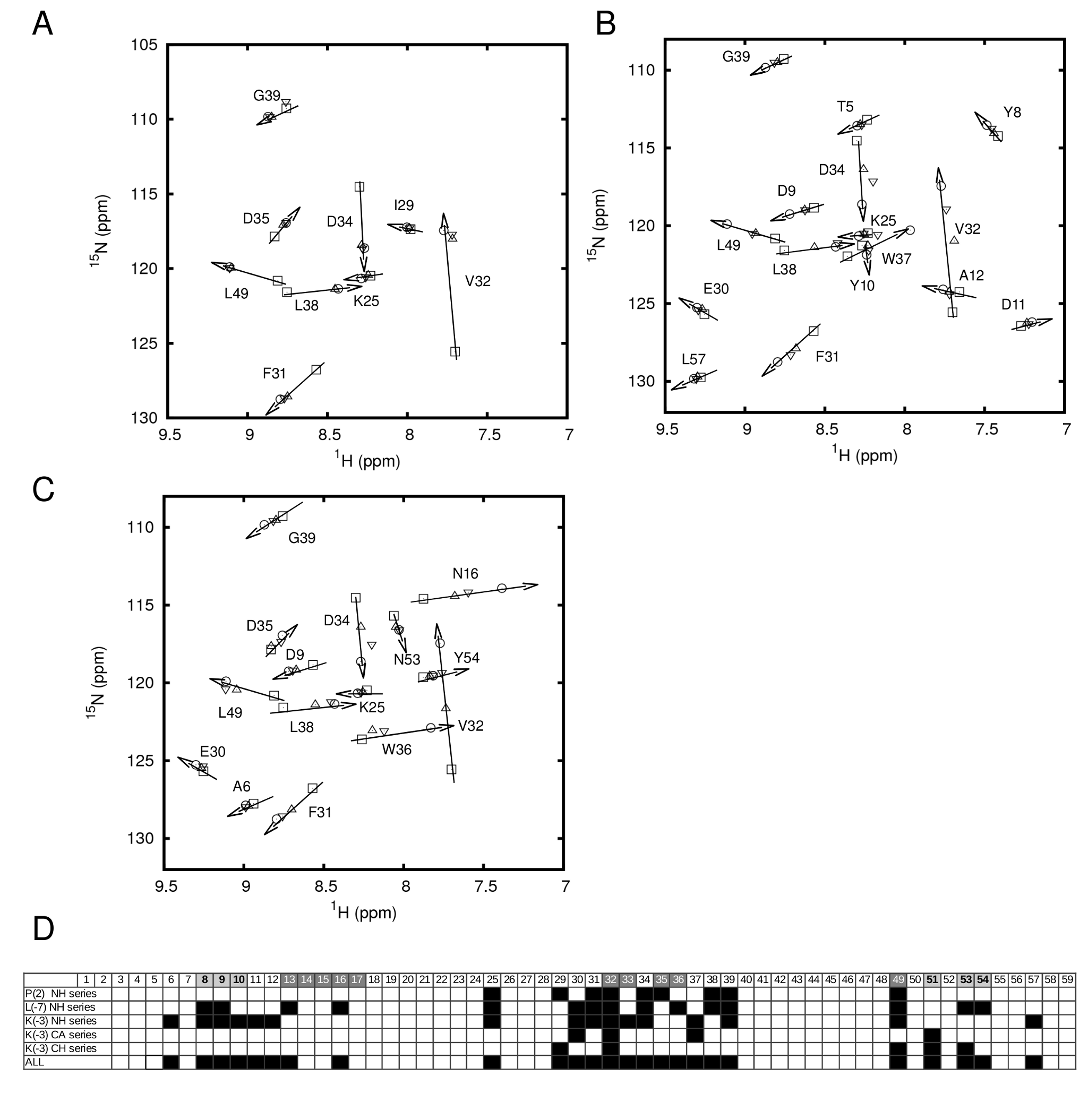

Supplement: Figure S2 — CCSP data for the P(2), K(−3) and L(−7) peptide series designed to minimize peptide sequence contamination. In order to minimize “contamination” by direct effects of different target peptide sequences, we re-probed the CCSP behavior of AbpSH3 complexes with ArkA-based peptides that would be hypothesized to display a range of SI or SII binding through point mutations of the three most energetically important ArkA positions, P(2), K(−3) and L(−7) (see highlighted positions in Fig. 2A inset, Table S3 and Table S4). Three different series were generated to minimize sequence specific “contamination”. Superposition of NH correlation spectra for complexes from the three series shows that many additional residues display CCSP behavior (Fig. S2D), including nearly all SI and SII residues and most residues close in space to SII. (A) CCSP behavior is found for 9 AbpSH3 residues in the P(2) series (representing a range of surface I binding [WT ArkA, P(2)A and P(2)V]) as seen in an overlay of 4 HSQC spectra (peak positions are represented by squares for peptide-free, up-triangle for “A” mutant, down triangle for “V” mutant and circle for ArkA-bound). The mutations were made in the SI-binding region of the peptide leading to contamination effects on the SI amides (that have smaller chemical shift changes, Table S1), however, CCSP behavior was seen in SII residues, providing evidence that SI and II are linked via conformational coupling. (B) CCSP behavior is found for 16 AbpSH3 residues in the K(−3) series (probing the boundary of surfaces I and II [WT ArkA, K(−3)A and K(−3)V], including those in both SI and SII). This result is expected as the site of mutation is close to both surfaces. (C) CCSP behavior is found for 15 AbpSH3 residues in the L(−7) series (representing a range of surface II binding [WT ArkA, L(−7)A and L(−7V)]). While the mutations were made in the SII binding region of the peptide, CCSP behavior is seen in SI residues, providing more evidence that SI and SII are [file pone.0051282.s002.tif]

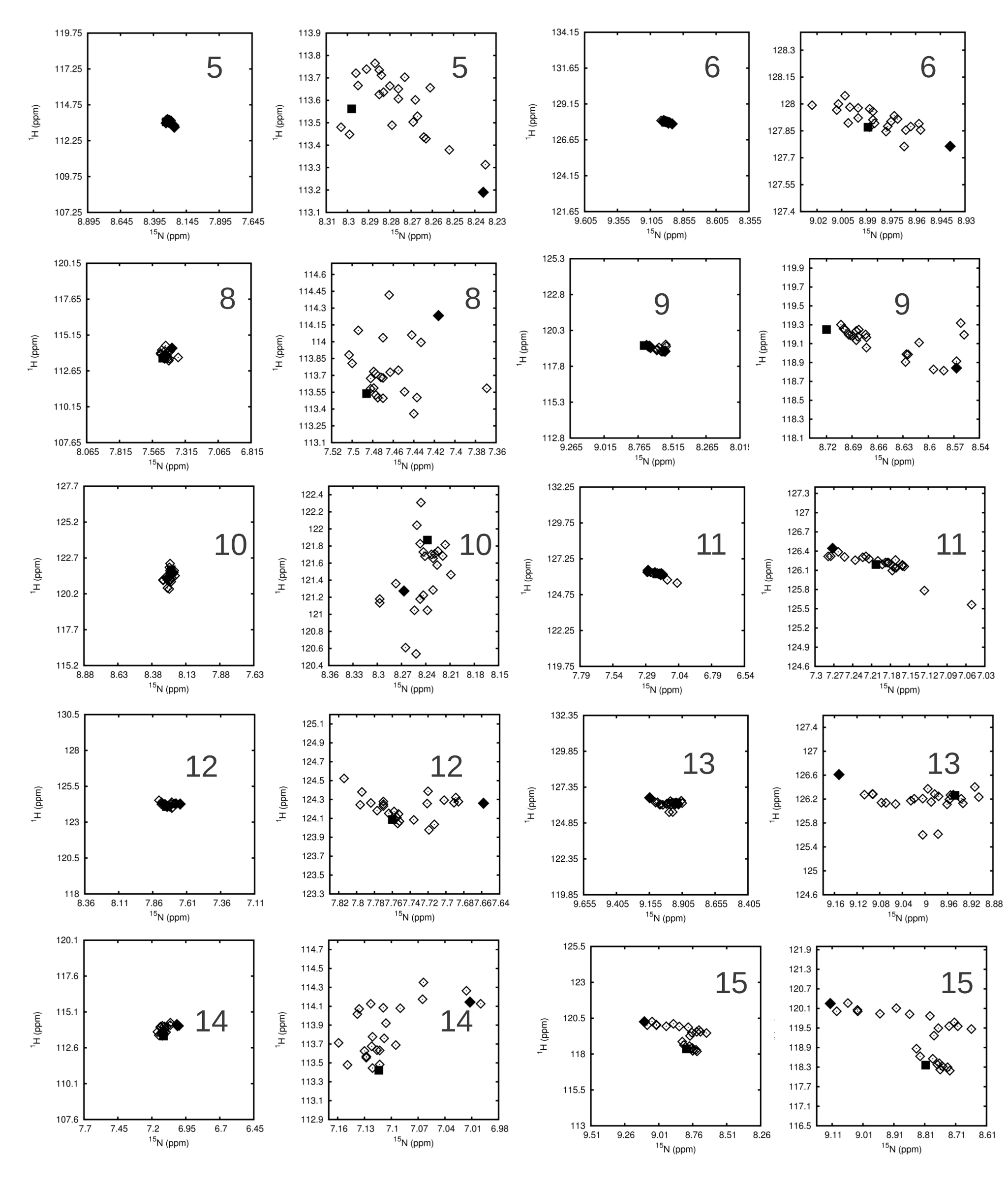

Supplement: Figure S3 — Overlay of NH correlation spectra of all 24 AbpSH3 complexes for residue resonance sets (5–15) with NH f- to -a shifts of 0.03 ppm or greater. The identity of the NH group plotted is indicated by the residue number inside the plot. All 24 complexes and the peptide-free AbpSH3 are included in these plots, where the peptide-free AbpSH3 (f conformer) is indicated by a filled diamond and the ArkA-bound AbpSH3 (a conformer) is indicated by the filled square. The first plot for each set is plotted at the same scale for all residues, while the axes in second plot are chosen to show the data more clearly. The error of a resonance position is calculated as ±0.0051 ppm for H and ±0.051 ppm for N. (TIF) [file pone.0051282.s003.tif]

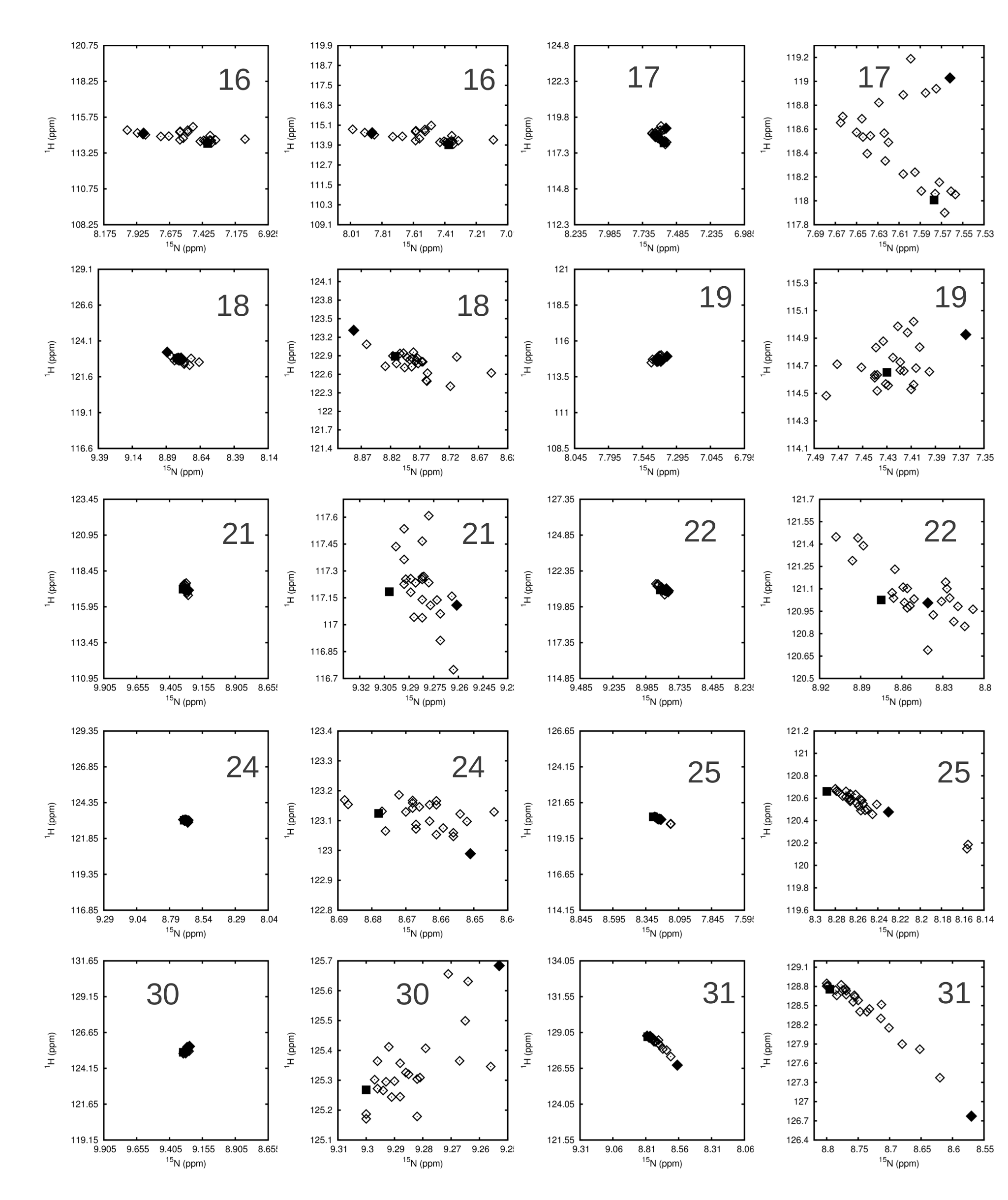

Supplement: Figure S4 — Overlay of NH correlation spectra of all 24 AbpSH3 complexes for residue resonance sets (16–31) with NH f- to -a shifts of 0.03 ppm or greater. The identity of the NH group plotted is indicated by the residue number inside the plot. All 24 complexes and the peptide-free AbpSH3 are included in these plots, where the peptide-free AbpSH3 (f conformer) is indicated by a filled diamond and the ArkA-bound AbpSH3 (a conformer) is indicated by the filled square. The first plot for each set is plotted at the same scale for all residues, while the axes in second plot are chosen to show the data more clearly. The error of a resonance position is calculated as ±0.0051 ppm for H and ±0.051 ppm for N. (TIF) [file pone.0051282.s004.tif]

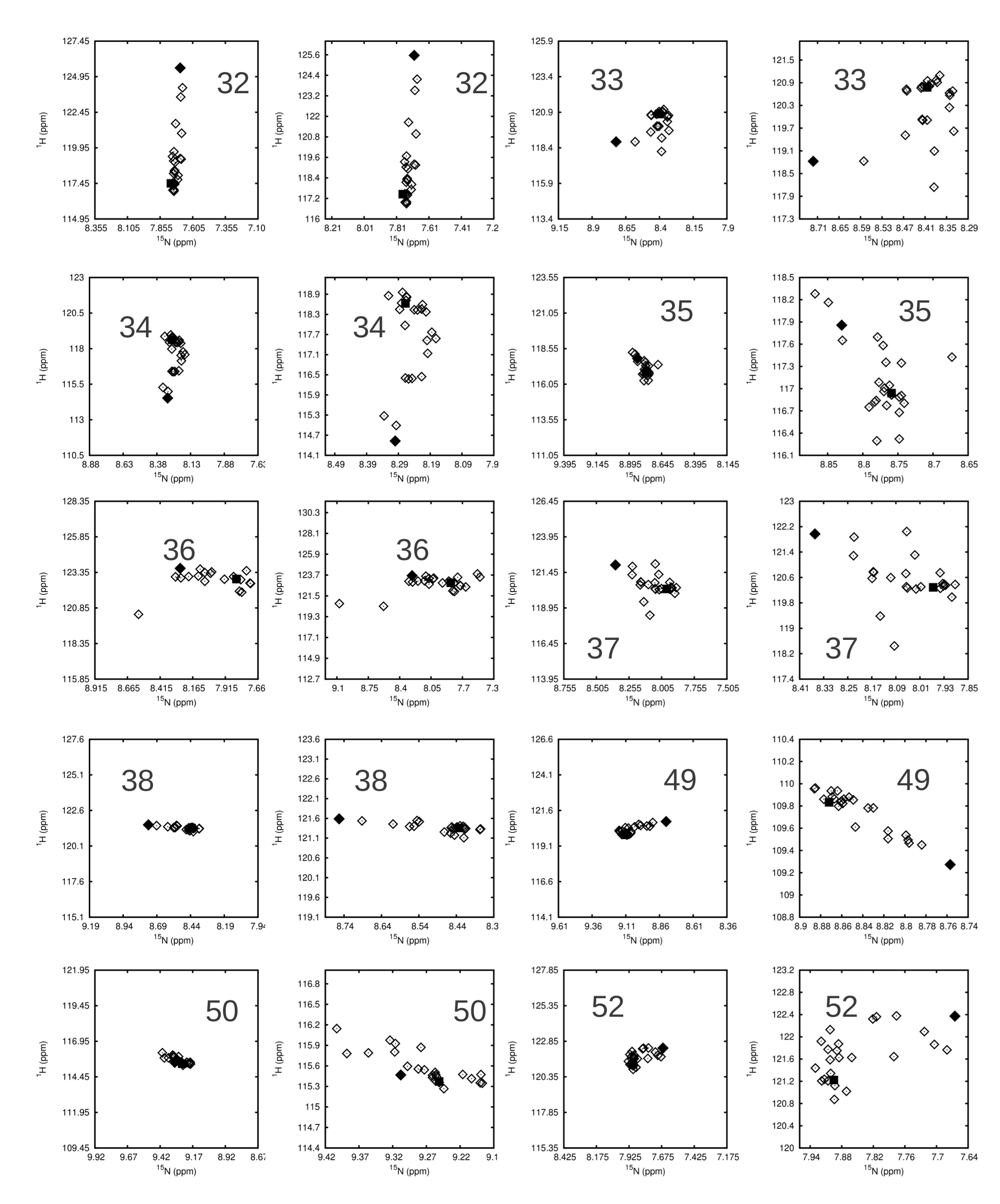

Supplement: Figure S5 — Overlay of NH correlation spectra of all 24 AbpSH3 complexes for residue resonance sets (32–52) with NH f- to -a shifts of 0.03 ppm or greater. The identity of the NH group plotted is indicated by the residue number inside the plot. All 24 complexes and the peptide-free AbpSH3 are included in these plots, where the peptide-free AbpSH3 (f conformer) is indicated by a filled diamond and the ArkA-bound AbpSH3 (a conformer) is indicated by the filled square. The first plot for each set is plotted at the same scale for all residues, while the axes in second plot are chosen to show the data more clearly. The error of a resonance position is calculated as ±0.0051 ppm for H and ±0.051 ppm for N. (TIF) [file pone.0051282.s005.tif]

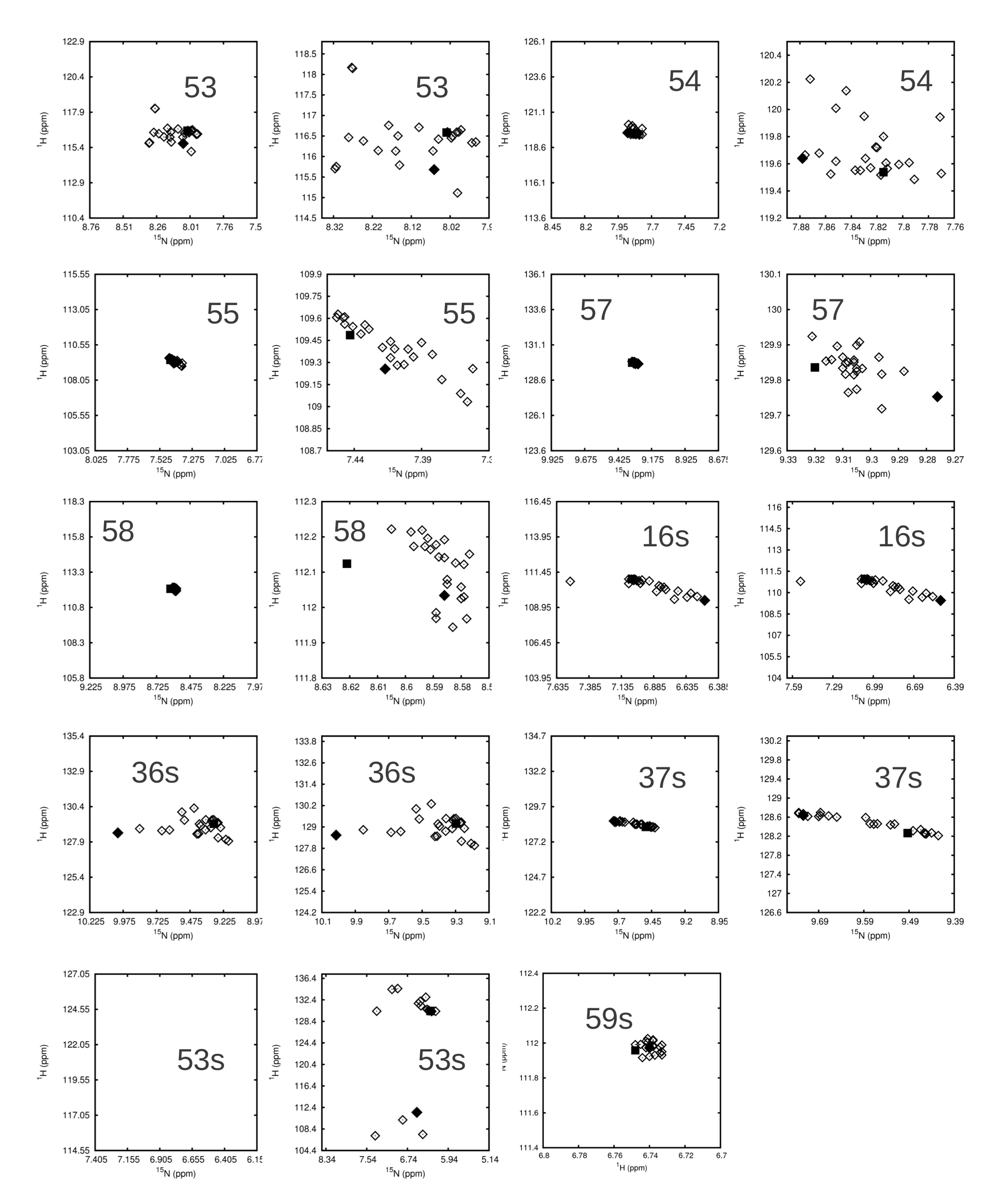

Supplement: Figure S6 — Overlay of NH correlation spectra of all 24 AbpSH3 complexes for residue resonance sets (53–58 and all side-chain amides) with NH f- to -a shifts of 0.03 ppm or greater. The identity of the NH group plotted is indicated by the residue number inside the plot. All 24 complexes and the peptide-free AbpSH3 are included in these plots, where the peptide-free AbpSH3 (f conformer) is indicated by a filled diamond and the ArkA-bound AbpSH3 (a conformer) is indicated by the filled square. The first plot for each set is plotted at the same scale for all residues, while the axes in second plot are chosen to show the data more clearly. The error of a resonance position is calculated as ±0.0051 ppm for H and ±0.051 ppm for N. (TIF) [file pone.0051282.s006.tif]

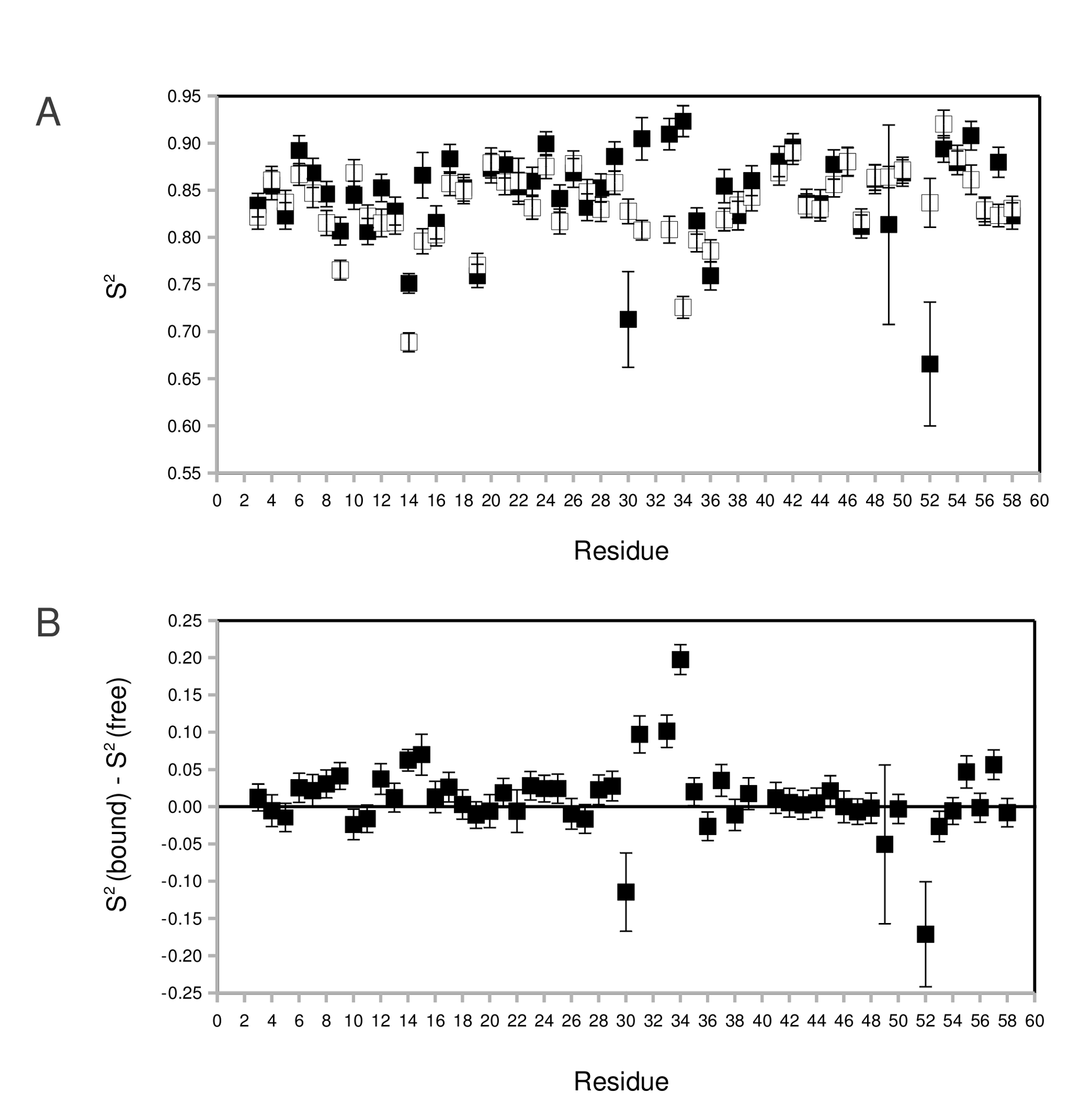

Supplement: Figure S7 — Backbone dynamics of the free and peptide-bound AbpSH3 (A) Order parameters calculated for the peptide-free (filled squares) and ArkA-bound AbpSH3 (open squares). See Protocol S1 for experimental details. (B) Difference plot of order parameters. The largest changes occur in the binding surface with a lesser effect seen in the residues associated with surfaces I and II. An Rex term (which is indicative of ms-µs conformational exchange) could be fit to data for 5–6 resonances in the peptide-free state that include the residues associated with the binding surface suggesting there is an exchange of conformations in the absence of peptide. Overall, the bound state is more rigid than the peptide-free state as can be seen in this difference plot (17 residues increase S2, 3 residues decrease S2), where the largest statistically significant changes are found for residues 33 and 34 in the N-Src loop which is part of SII. Three residues (30, 49, 52) appear to increase flexibility upon binding, although there are large errors associated with these residues. The Rex term could not be fit to data for any residue in the bound state, although a faster timescale term, te was fit for residue 14, 15, and 16. Taken together, peptide binding reduces the backbone dynamics in AbpSH3 in both the ns-ps and ms-µs timescale regimes, involving residues in and around surface I and II. As noted here and in our previous studies, peptide binding does not completely dampen the dynamics in the tip of the RT- loop (residues 14, 15, 16). (TIF) [file pone.0051282.s007.tif]

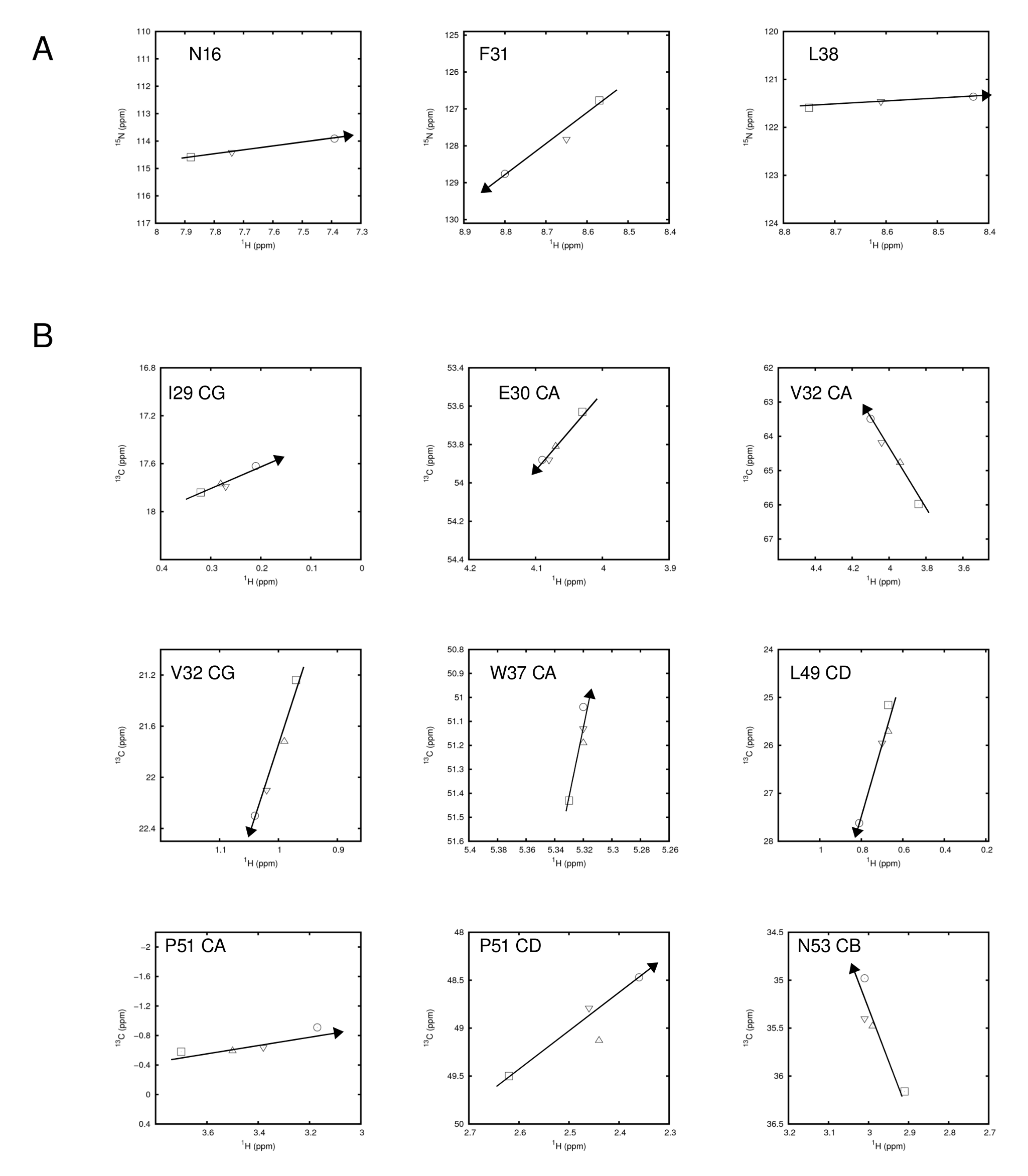

Supplement: Figure S8 — Further evidence for protein conformational effects within the dynamic bound-state ensemble of AbpSH3:peptide complexes. (A) CCSP behavior is seen in and around SII for the complex with a 10-residue primarily SI-binding peptide from ArkA. Three examples are shown for residues L38, F31 and N16. All of these residues are far from the SI-binding site, suggesting that SI and SII are connected through conformational coupling. The labels are as follows; square is peptide-free AbpSH3, down-triangle is SI-binding peptide complex and circle is ArkA complex. (B) CCSP behavior found for aliphatic C-H correlation peaks in the K(−3) peptide series. Overlay of spectra (peak positions are represented by squares for peptide-free, up-triangle for “A” mutant, down triangle for “V” mutant and circle for ArkA-bound) highlighting CCSP behavior for CA-HA correlations and side-chain C-H correlations. The CCSP values from this analysis confirm P51 undergoes conformational exchange (which could not be determined from previous experiments that only probed amide groups) and revealed a general correlation with their amide groups. It should be noted that significant spectral crowding and overlap of aliphatic resonances made it difficult to identify as many examples of CCSP behavior for CH groups. (TIF) [file pone.0051282.s008.tif]

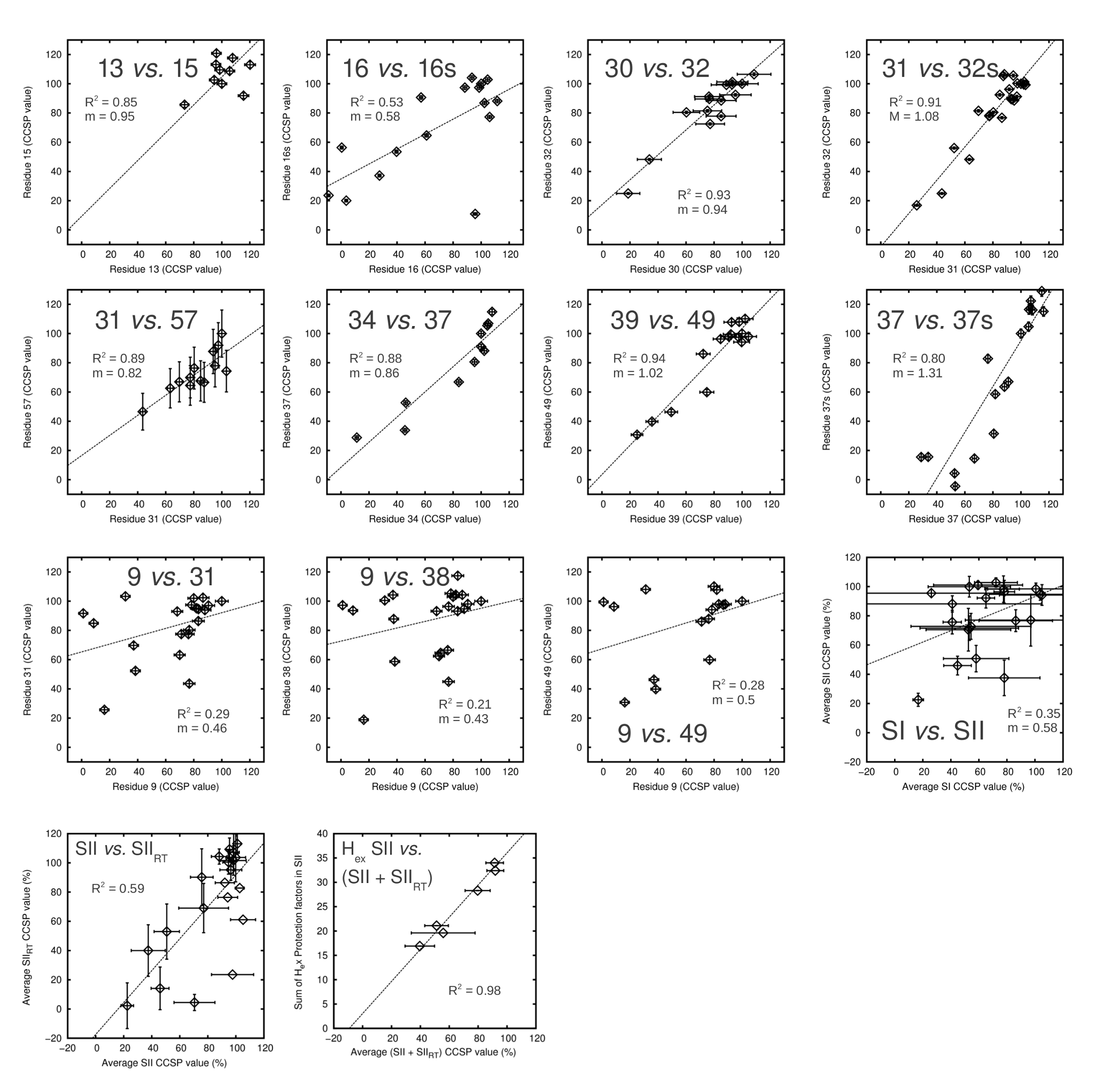

Supplement: Figure S9 — Supporting data for CCSP analysis. CCSP pairwise correlation plots. m is the slope of the best fit line. The outliers in the SII vs. SIIRT plot were from ArkA K(−3)A, ArkA K(−3)V, Scp12 and Scp17 suggesting the tip of the RT loop is contaminated in these complexes. (TIF) [file pone.0051282.s009.tif]

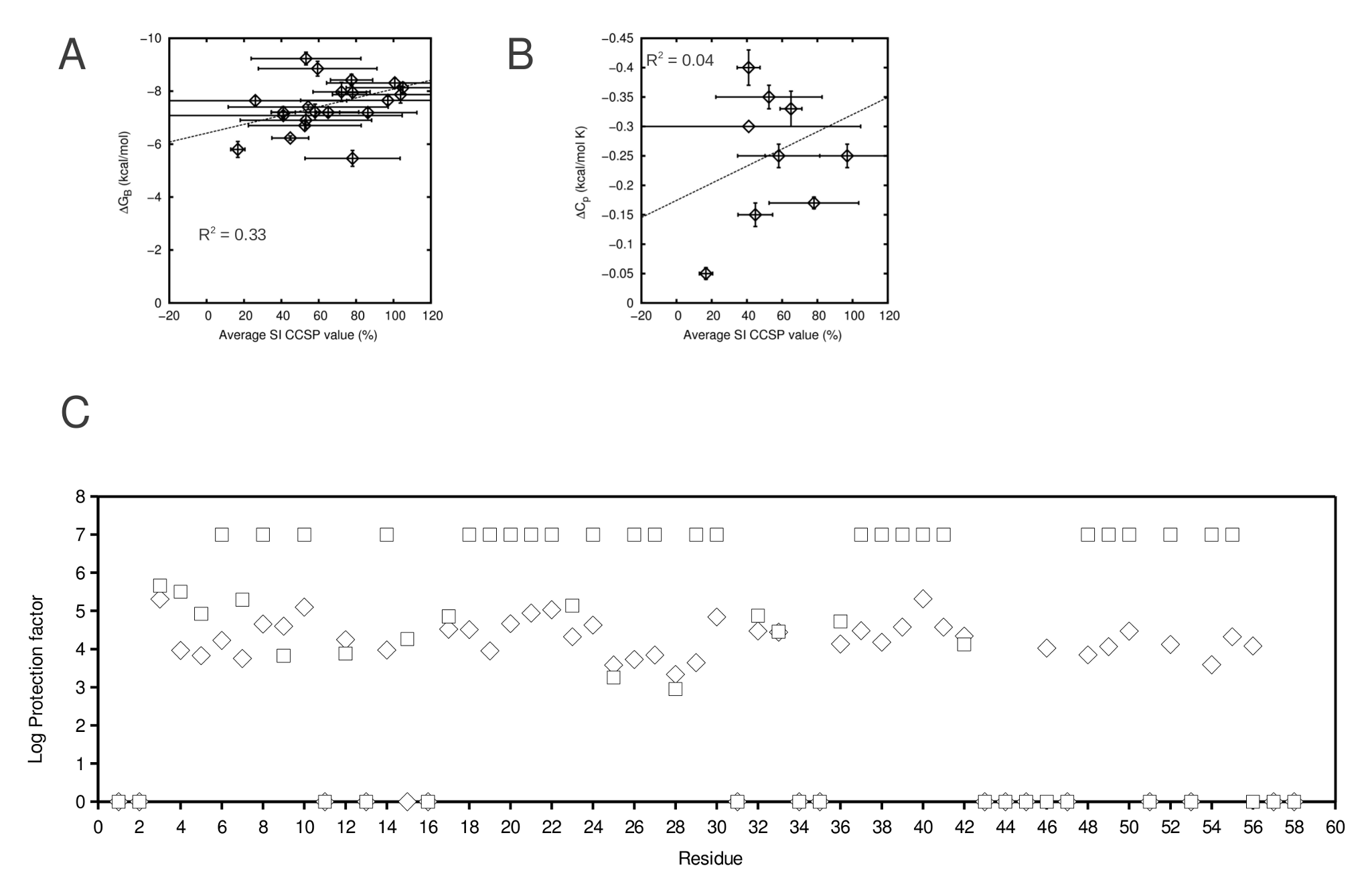

Supplement: Figure S10 — Correlation of thermodynamic parameters with average CCSP value for SI. (A) Average CCSP value for SI vs. ΔGB. (B) Average CCSP value for SI vs. ΔCp. Data are mean ±95% confidence intervals except the ΔCp values which report the standard error of the least squared fit to the data (ΔH vs. temperature). (C) Hydrogen exchange protection data for peptide-free and ArkA-bound AbpSH3. Hydrogen exchange protection factors obtained for peptide-free AbpSH3 (diamonds) and ArkA bound AbpSH3 (squares). Protection factor values of 0 indicate residues exchanging at rates that are too fast to measure, and protection factor values of 7 indicate residues exchanging at rates that are too slow to determine accurately or did not undergo observable exchange. Several of the squares and diamonds are overlapping with a value of 0. (TIF) [file pone.0051282.s010.tif]

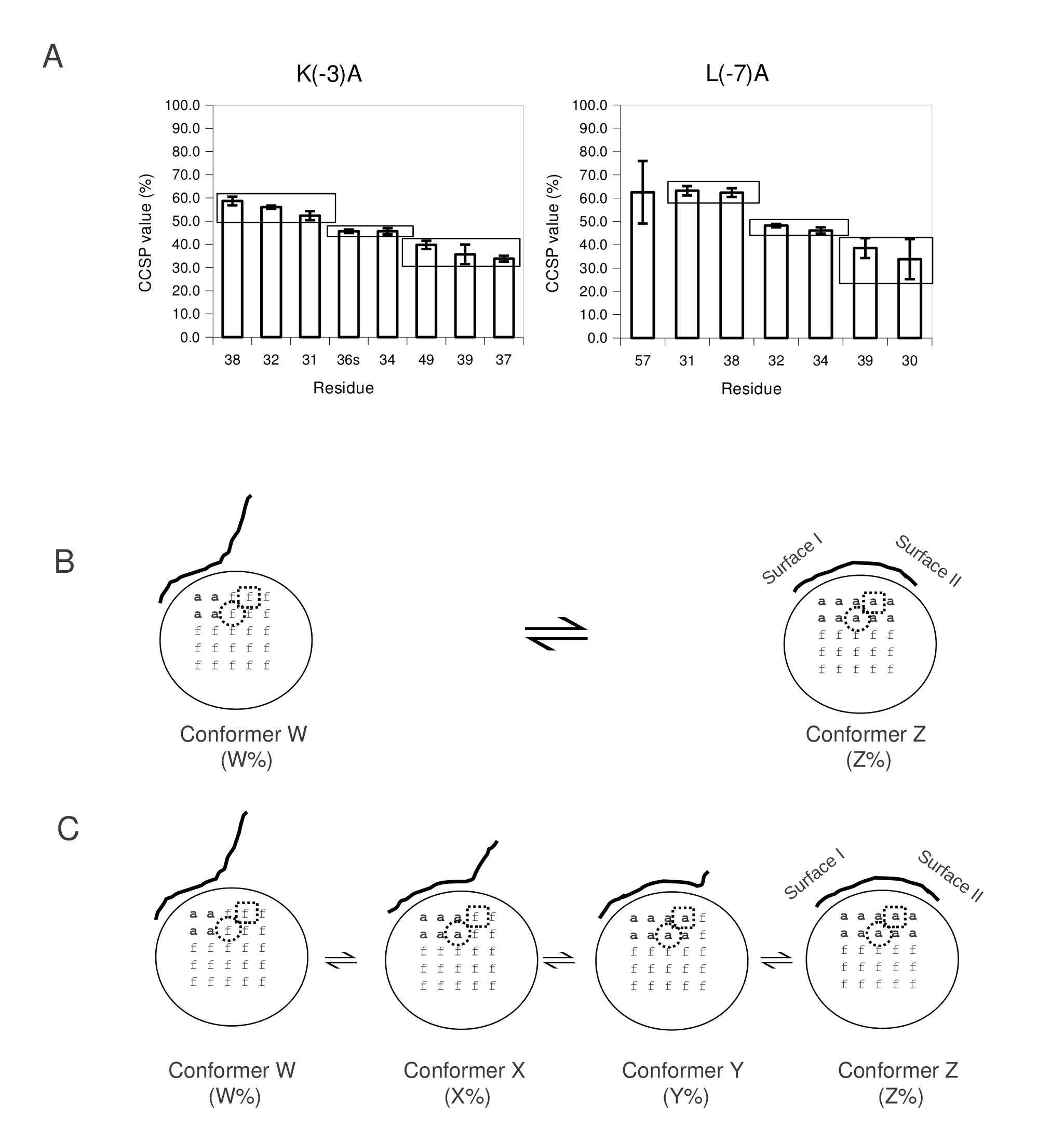

Supplement: Figure S11 — Variability in CCSP values within the complexes. (A) Individual SII residue CCSP values for 2 ArkA mutant complexes, the error bars are 95% confidence intervals and the boxes reveal at least three residues with statistically different CCSP values (non-overlapping error bars). (B) A possible 2 macro-state model. Two residues are considered (broken circle and square) in an ensemble with only two exchanging protein conformers (W and Z conformations), which are populated W% and Z%, respectively. In this ensemble the CCSP value for both residues would be Z% as both residues are only in the a residue-level conformer in the Z protein conformer. (C) An alternative multi-macro-state model. The same two residues are considered in an ensemble with four exchanging protein conformers (W, X, Y and Z conformations), which have the population ratio W∶X∶Y∶Z. In this ensemble the CCSP value for the residue highlighted by a square would be (Y+Z)% as it is only in the a residue-level conformer in the Y and Z protein conformer. However the residue highlighted by the circle will have a CCSP value of (X+Y+Z)% because it is in the a residue-level conformer in the X, Y and Z protein conformers. By having more than two protein conformers that have different compositions of f and a, individual residues throughout the domain are able to show a range of different CCSP values because the combination of protein conformers where one particular residue is in the a residue-level conformer is likely to be different to the combination of protein conformers where another residue is in the a residue-level conformers. The behavior of the peptide that is presented in this model is only one of many possible scenarios (see Fig. S12). (TIF) [file pone.0051282.s011.tif]

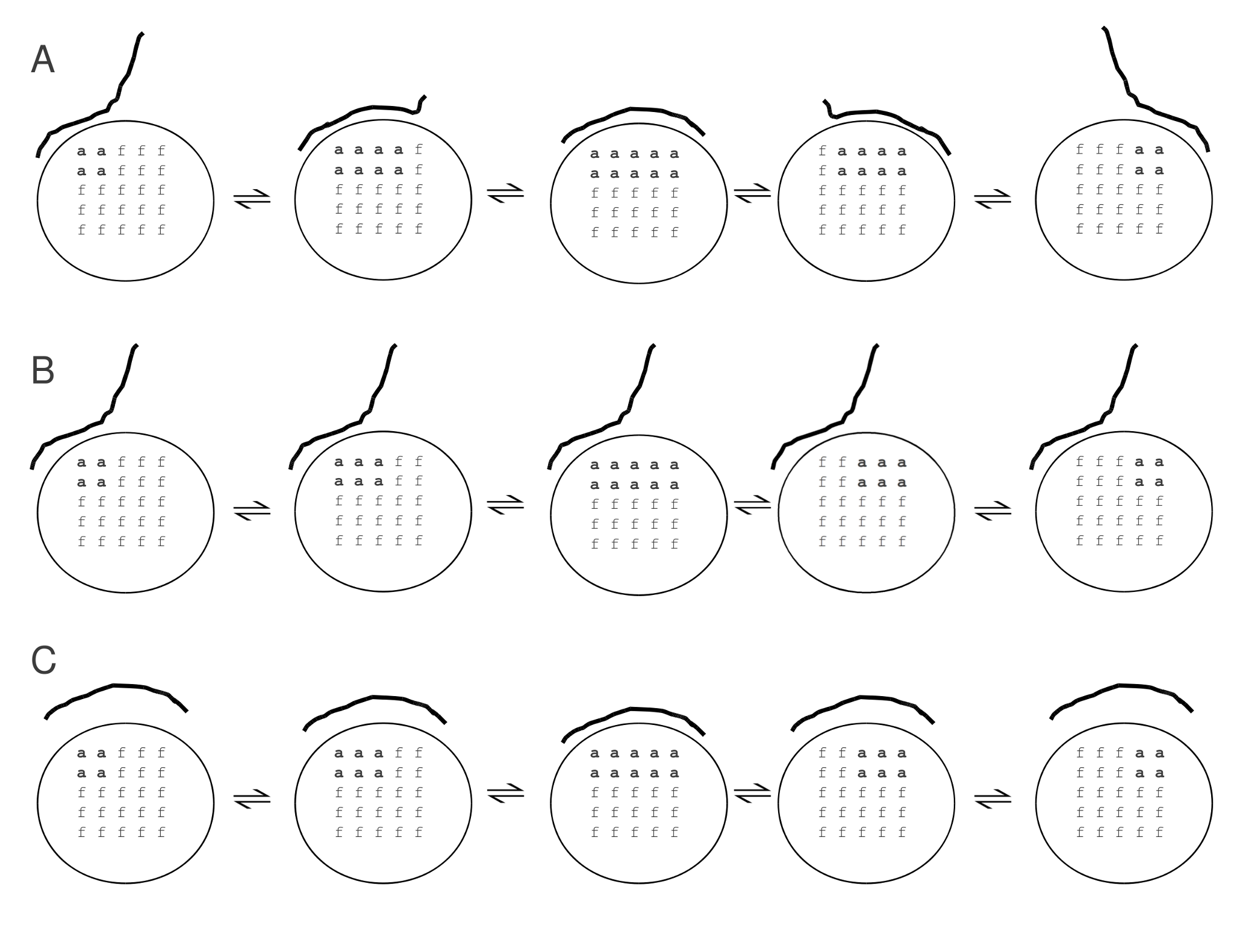

Supplement: Figure S12 — Possible models for the AbpSH3:peptide ensemble. (A) Protein residues undergo f-to-a exchange as the physical association of the peptide increases then decreases across the binding surface. (B) Protein residues undergo f-to-a exchange as a conformational response to partial binding of the peptide to part of the binding surface. (C) Protein residues undergo f-to-a exchange as peptide that is physically associated to the complete binding surface becomes more tightly engaged then less tightly engaged, for example by progressively expelling/incorporating waters or counter ions at the interface or refinement of side-chain packing at the interface. The actual ensemble may also be any combination of these models. (TIF) [file pone.0051282.s012.tif]

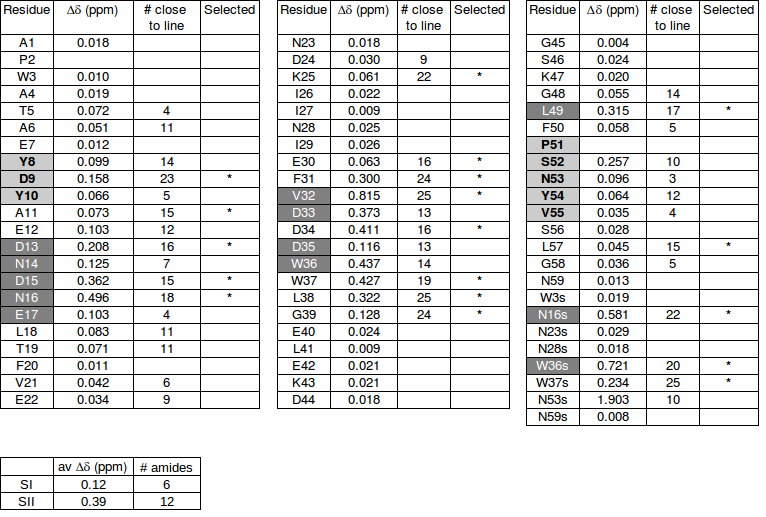

Supplement: Table S1 — Summary of data used to screen amide groups that were significantly contaminated by the direct effects of peptide sequence differences. Δδ is the combined chemical shift difference between the resonance of the amide in the peptide-free AbpSH3 and the corresponding resonance in the ArkA-bound AbpSH3. # close to the line refers to the number of resonances in any given residue set that has perpendicular distances that are less than 10% of its CCSP line. Those residues that have a δΔ of 0.03 ppm or greater and belong to a set that has 60% or greater perpendicular distances that are less than 10% of its CCSP line were deemed most likely free of contamination and chosen for further analysis (indicated by a star in the fourth column). Residue numbers followed by “s” refer to the side-chain NH group. SI residues are highlighted in red and SII residues in blue. The small table below the main table, indicates the number of residues that have a Δδ of 0.03 ppm or greater in each surface, as well as its average value. SI data is much more limited in this type of analysis, due to fewer residues involved in chemical shift changes and with much smaller magnitude compared to SII. (TIF) [file pone.0051282.s013.tif]

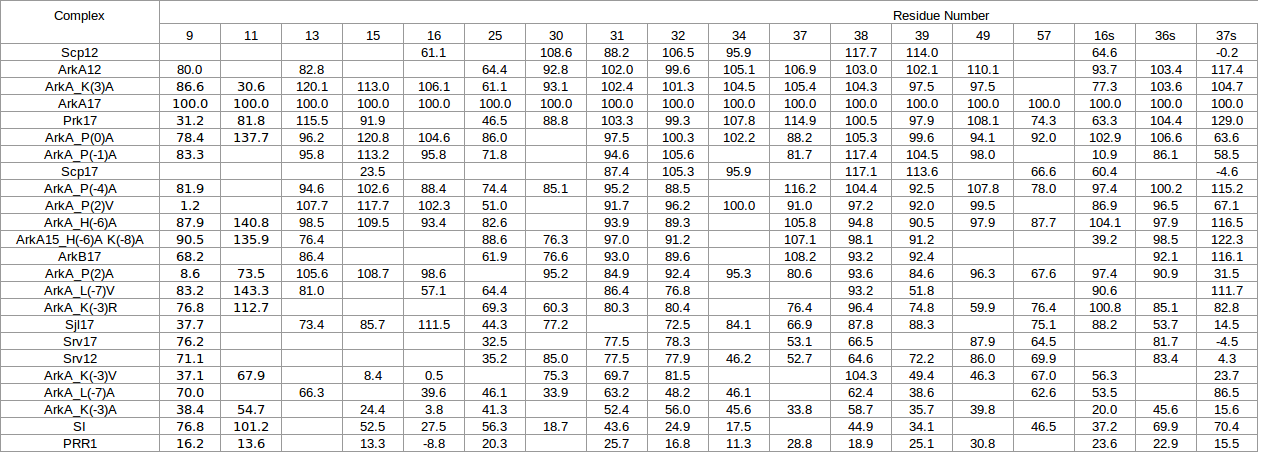

Supplement: Table S2 — CCSP values for all residues analyzed further in this study. As mentioned in Table S1, several SI-associated residue values are missing due to the fact that chemical shift changes were much smaller in this region compared to SII which allows for greater contamination effects. Residue numbers followed by “s” refer to the side-chain NH group. (TIF) [file pone.0051282.s014.tif]

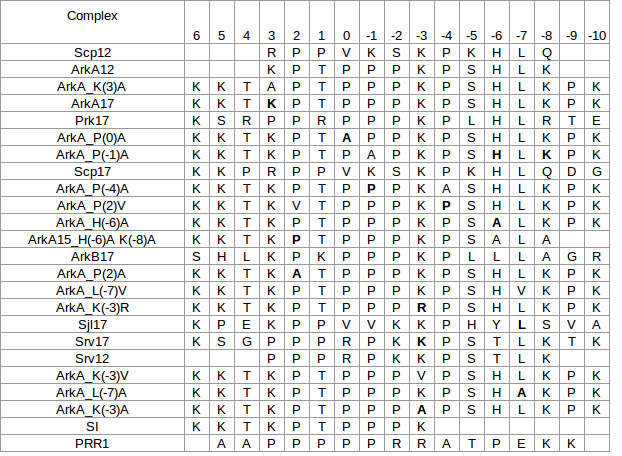

Supplement: Table S3 — Alignment of sequences used in this study. Residues in bold are mutation sites. The sequences are ordered according to their average CCSP values in SII. (TIF) [file pone.0051282.s015.tif]
