# Supplementary material for: Differential Dynamic Engagement within 24 SH3 Domain: Peptide Complexes Revealed by Co-Linear Chemical Shift Perturbation Analysis
Source: PLoS One. 2012 Dec 12;7(12):e51282. doi: 10.1371/journal.pone.0051282 (PMC3520974; doi:10.1371/journal.pone.0051282)
Supplement: Protocol S1 — (DOCX) [file pone.0051282.s017.docx]

**Protocol S1. NMR relaxation**

Decreases in peak volume as function of relaxation delay was calculated for the ^15^N T_1_ and T_2_ relaxation rates for peptide free AbpSH3 or AbpSH3 with unlabeled ArkA bound. Rate errors were estimated using deviations of the experimental values from the best-fit decay curve (1). Heteronuclear NOEs for peptide free AbpSH3 or AbpSH3 with unlabeled ArkA bound were calculated as previously described (1). Errors were estimated from spectral noise.

The program DASHA 4.1 (2) was used for the model-free analysis of the relaxation data. The parameters were fit to three different models using an anisotropic rotation diffusion tensor, Model 1 used S^2^ only, Model 2 used S^2^ and t_e_ and Model 3 used S^2^ and R_ex_. Residues were excluded in the analysis if ^1^HN-^15^N NOE values were less than 0.7. Each model was applied in turn and each residue was assigned a model based on chi-squared and F-test values, similar to the approach of Mandel and colleagues (3).

**References**

1. Farrow N A, Muhandiram R, Singer A U, Pascal S M, Kay C M, Gish G, Shoelson S E, Pawson T, Forman-Kay J D, Kay L E (1994) Backbone dynamics of a free and phosphopeptide-complexed Src homology 2 domain studied by 15N NMR relaxation. Biochemistry 33:5984–6003

2. Nolde, D.E., Golovanov, A.P., Korzhnev, D.M., Arseniev, A.S. (1995) Processing of heteronuclear NMR relaxation data with the new software DASHA. Appl. Magn. Reson. 9:581-588

3. Mandel AM, Akke M, Palmer AG III (1995) Backbone dynamics of Escherichia coli ribonuclease HI: correlations with structure and function in an active enzyme. J Mol Biol 246:144–163

4. Maxwell, K. L., and Davidson, A. R. (1998) Mutagenesis of a buried polar interaction in an SH3 domain: sequence conservation provides the best prediction of stability effects. *Biochemistry 37, 16172 16182*
